# Evaluation of Electromagnetic Properties of Fe–5.0 wt.%Si According to MoS_2_ and Graphite Lubricant Contents

**DOI:** 10.3390/ma19204311

**Published:** 2026-10-13

**Authors:** Jehyeon Park, Seonbong Lee

**Affiliations:** 1 Department of Mechanical Engineering, Keimyung University, Daegu 42601, Republic of Korea; 5648967@stu.kmu.ac.kr; 2 Department of Automotive Engineering, Keimyung University, Daegu 42601, Republic of Korea

**Keywords:** soft magnetic composites, Fe–Si alloy, MoS_2_, graphite, lubricant compaction, core loss, coercivity, relative density, interfacial characteristics

## Abstract

Soft magnetic composites (SMCs) require a balance between internal density uniformity, microstructure, and electromagnetic performance, all of which are affected by lubricant composition. This study investigated Fe–5.0 wt.%Si SMC toroidal cores with nine MoS_2_/graphite compositions using FEA, electromagnetic measurements, SEM, and TEM–EDS. C3, containing 1.0 wt.% MoS_2_ and 0.3 wt.% graphite, exhibited the smallest relative-density Max–Min (0.439) and the highest relative-density Min (0.558). Its comparatively low powder–die friction reduced frictional resistance to load transmission and limited local low-density development near the inner die-contact boundary, resulting in a more uniform internal relative-density distribution. At 300 mT, C3 showed the lowest *P*_cv_ values of 113.50 and 173.70 kW/m^3^, *P*_cm_ values of 15.54 and 23.78 W/kg, and *H*_c_ values of 134.83 and 137.40 A/m at 1000 and 1500 Hz, respectively. SEM showed relatively uniform interparticle contact across the specimen height, while TEM–EDS revealed thin and similar interfacial regions in two C3 specimens. Together with the low graphite content, these densification and interfacial characteristics were associated with the lowest core loss. Overall, C3 showed the best combined performance among the nine compositions.

## 1. Introduction

Soft magnetic composites (SMCs), unlike conventional laminated electrical steels, allow three-dimensional magnetic flux paths and can suppress eddy-current loss because of their high electrical resistivity [1,2]. This three-dimensional design freedom can also improve volumetric utilization when the motor topology is specifically designed to exploit the geometrical flexibility of SMCs. For example, in a topology-specific comparison, Lim et al. reported torque per total volume values of 10.6 kNm/m^3^ for an SMC machine with embedded stator end-windings and 9.9 kNm/m^3^ for the corresponding manufacturable non-embedded laminated silicon–steel machine, corresponding to an approximately 7.1% increase for the embedded SMC configuration [3]. These characteristics have promoted the use of SMCs as core materials for electric motors, inductors, and other electromagnetic devices [1,2]. Among various SMC materials, Fe–Si alloys are used as motor-core materials because of their high saturation magnetization and the increase in electrical resistivity associated with Si addition [4]. Increasing the Si content generally improves electrical resistivity and can suppress eddy-current loss, although it may also reduce ductility and formability. In the present study, Fe–5.0 wt.%Si was selected to maintain consistency with the previous investigation of MoS_2_/graphite lubricant compositions [5] and to evaluate their effects on electromagnetic properties without changing the base alloy composition. However, the electromagnetic properties of Fe–Si SMCs are determined by material and microstructural factors, including the composition of the starting powder, compact density and density uniformity, interparticle insulation, residual stress, pores, and interparticle interface conditions [4,6,7].

In powder-metallurgy-based SMC manufacturing, lubricants are added to reduce friction between powder particles and at the powder–die interface during compaction [8,9]. Lubricant addition affects powder rearrangement and the compaction process, which can change the final density and internal density distribution of the compact [8,9,10]. The type and amount of lubricant also affect the ejection process and the mechanical integrity of the compact [8,9]. MoS_2_ acts as a solid lubricant owing to its layered crystal structure and low shear resistance between adjacent basal planes, which facilitates interfacial sliding. Previous tribological studies have also shown that MoS_2_ particles can form friction-reducing interfacial films at contacting surfaces [11,12]. In dry powder compaction, the tribological role of MoS_2_ is primarily associated with solid-state interfacial contact, adhesion, sliding, and shear behavior. Graphite also provides solid lubrication through its layered structure and can facilitate interparticle sliding during compaction [13]. However, graphite is electrically conductive, and excessive or locally continuous graphite-rich regions can reduce the effective electrical resistance between magnetic particles. This may increase the contribution of eddy-current loss when interparticle electrical isolation is degraded [4,14]. Therefore, the graphite content should be considered in terms of both its tribological contribution during compaction and its possible influence on subsequent electromagnetic performance.

The effects of lubricants also need to be considered after the compaction stage [5,7,15]. Depending on the type and content combination of the lubricants, differences can occur in pores and interparticle contact conditions after compaction, and differences can also occur in the interparticle interface structure and the local distribution of lubricant-derived elements after heat treatment [5,7,15,16,17]. These microstructural differences can be related to interparticle interface conditions and electromagnetic properties, including magnetic loss characteristics such as core loss [6,7,15]. Therefore, both the compaction state and microstructure need to be considered when evaluating electromagnetic properties according to lubricant composition [5,6,7,15].

Finite-element and numerical studies of powder compaction have identified powder–die friction as an important factor governing stress transmission and density gradients within pressed compacts. Briscoe and Rough demonstrated experimentally and numerically that changes in die-wall friction altered the stress and density distributions generated during powder compaction [10]. More recent FEM studies have also shown that tool geometry and powder–die interaction conditions affect local densification during pressing [18]. Khoei et al. further reported that die-wall friction influences relative-density evolution, stress–strain response, and pressure distribution during powder compaction [19]. These studies indicate that the friction coefficient is an important boundary condition when FEA is used to predict the internal compaction state. However, studies that apply lubricant-composition-specific friction conditions derived from experimental tribological data to SMC compaction under common load stop conditions and subsequently relate the predicted internal density distribution to electromagnetic performance remain limited.

Recent SMC studies have examined the effects of manufacturing variables, including compaction conditions, lubricant and binder contents, and insulation-layer composition, on compact density and magnetic properties [6,7,15]. Huang et al. analyzed changes in compact density and soft magnetic properties according to compaction temperature, compaction pressure, lubricant content, and binder content during warm compaction of FeSiCr SMCs [20]. Yang et al. investigated the effect of zinc stearate content on the microstructure and soft magnetic properties of Fe-based nanocrystalline SMCs and reported that the lubricant content affected residual stress, permeability, and core loss [21]. Motozuka et al. investigated lubricant oil and boron nitride as milling aids for iron powders used in SMCs and showed that the type of lubricant influenced recrystallization behavior and the resulting permeability and coercivity [22]. Xie et al. investigated kaolin-containing hybrid coatings in Fe-based nanocrystalline SMCs and showed that an appropriate kaolin content acted as a solid lubricant during compaction, affecting particle packing, coating integrity, and core-loss characteristics [23]. Zhu et al. analyzed permeability and magnetic loss in FeSiAl/MoS_2_ composites using MoS_2_ as an insulating component [24], and a subsequent study examined the effects of particle size and transverse magnetic field on the high-frequency magnetic properties of FeSiAl/MoS_2_ [25]. Recent studies in particulate and nanoscale lubrication have additionally shown that tribological performance can depend strongly on additive concentration and interfacial conditions [26]. More directly for powder pressing, studies of MoS_2_-containing powders have shown that solid-particle lubrication can influence densification, while excessive MoS_2_ addition may cause particle agglomeration and reduce powder compressibility [9]. These findings indicate that lubricant content should be treated as a controlled process variable rather than assuming that lubrication continuously improves with increasing additive content.

In our previous study, the same nine MoS_2_/graphite composition conditions were evaluated primarily in terms of high-temperature friction behavior, compaction behavior, hardness distribution, FEA-based densification behavior, and mechanical properties [5]. Electromagnetic properties were proposed as a subject for subsequent investigation [5]. The present study extends the previous mechanical investigation by newly evaluating the electromagnetic properties of all nine MoS_2_/graphite composition conditions and subsequently examining the composition-dependent microstructural characteristics using SEM for all nine cases and TEM–EDS for selected representative cases. A previous study on Fe–5.0 wt.%Si SMCs evaluated electromagnetic properties while varying H_3_PO_4_, polyimide, and MoS_2_ or graphite as additive factors [15]. In contrast, the present study maintains fixed H_3_PO_4_ and polyimide contents and systematically varies the simultaneous combination of MoS_2_ and graphite using a 3 × 3 composition matrix. Within the literature reviewed in this study, the combined evaluation of a controlled MoS_2_/graphite co-lubricant matrix, lubricant-composition-specific friction-based FEA, electromagnetic properties, and SEM/TEM–EDS interfacial characteristics has not been systematically reported for Fe–5.0 wt.%Si SMCs.

The FEA and electromagnetic property measurements in this study were performed for different but complementary purposes. In the FEA, the case-specific temperature-dependent powder–die friction conditions obtained from the previous tribological study [5] were applied under common load-stop conditions to evaluate how the differences in friction were reflected in the final compact height, relative-density distribution, and internal relative-density uniformity. C3, which denotes the composition containing 1.0 wt.% MoS_2_ and 0.3 wt.% graphite, was selected as the reference case for establishing the common load-stop conditions. In contrast, electromagnetic measurements were performed on the fabricated toroidal cores to directly evaluate their measured electromagnetic performance, including inductance, permeability, Q value, core loss, and coercive field. The FEA-derived compaction characteristics and the measured electromagnetic properties were subsequently examined together with SEM observations for all nine cases and TEM–EDS observations for selected representative cases to interpret the differences among the lubricant compositions from the perspective of internal densification, interparticle microstructure, and interfacial characteristics.

Accordingly, the significance of this study lies in establishing an integrated relationship among MoS_2_/graphite lubricant composition, case-specific powder–die friction conditions, FEA-based internal densification, measured electromagnetic performance, and interparticle microstructure under fixed insulation and binder conditions. By maintaining the H_3_PO_4_ and polyimide contents and the compaction and heat-treatment conditions, the influence of the MoS_2_/graphite co-lubricant composition can be evaluated separately from changes in other processing variables.

## 2. Materials and Methods

### 2.1. Powder and Lubricant Composition Conditions

In this study, the Fe–5.0 wt.%Si alloy powder used in the previous study was used as the starting material [5]. The powder composition was 94.85 wt.% Fe, 5.02 wt.% Si, and 0.13 wt.% O [5,15]. The particle size distribution was measured using a particle size analyzer (Mastersizer 3000, Malvern Instruments Ltd., Malvern, UK). The Dv(10), Dv(50), and Dv(90) values were 38.058, 76.247, and 141.126 μm, respectively, and the volume-weighted mean particle diameter, D [3,4], was 83.600 μm [5].

To maintain the same insulation and binder conditions for all specimens, the H_3_PO_4_ and polyimide contents were fixed at 0.25 wt.% each [5,6,15,27,28]. The Fe–5.0 wt.%Si powder was sequentially treated with H_3_PO_4_ and coated with polyimide according to the method described in the previous study [5,6,15,27,28]. MoS_2_ and graphite were then added as solid lubricants [5,13,29,30,31]. The particle sizes of the MoS_2_ and graphite powders were approximately 3 μm and less than 20 μm, respectively [5]. For each lubricant composition, the coated Fe–5.0 wt.%Si powder was manually mixed with the corresponding amounts of MoS_2_ and graphite using a mortar and pestle for 10 min. The powder mixture was then transferred to a plastic mixing container and mixed for an additional 5 min at an average rate of approximately 90 cycles/min. The same mixing procedure was applied to all nine cases [5].

The lubricant and binder composition conditions used in this study are summarized in Table 1. The MoS_2_ content was set at three levels of 0.5, 0.75, and 1.0 wt.%, and the graphite content was varied at three levels of 0.3, 0.5, and 0.8 wt.%, resulting in nine composition conditions [5,15]. In the case designations, A, B, and C correspond to MoS_2_ contents of 0.5, 0.75, and 1.0 wt.%, respectively, while the numbers 3, 5, and 8 correspond to graphite contents of 0.3, 0.5, and 0.8 wt.%, respectively [5]. This 3 × 3 composition design was used to compare the differences in FEA-based relative-density distribution, internal density uniformity, and electromagnetic properties of Fe–5.0 wt.%Si soft magnetic composites according to the combinations of MoS_2_ and graphite contents.

### 2.2. Compaction and Heat Treatment of Toroidal Specimens

Toroidal specimens were fabricated using powders with the nine MoS_2_/graphite lubricant composition conditions listed in Table 1 [5]. The powder charge for each specimen was set to 6.0 ± 0.1 g. The nominal geometry of the toroidal specimens in this study was defined as an outer diameter of 20.41 mm, an inner diameter of 12.57 mm, and a target height of 4.0 mm.

The specimens were fabricated using a 2-Press 1-Anneal (2P1A) process consisting of two pressing steps and one heat-treatment step [5]. For the first pressing, the die and powder were heated to 350 °C in an air atmosphere, and a press setting of 60 kgf, corresponding to the 50% condition of the specimen fabrication equipment, was applied [5]. For the second pressing, the die and compact were heated to 550 °C under an Ar gas flow atmosphere, and a press setting of 120 kgf, corresponding to the 100% condition of the equipment, was applied [5]. The temperature, atmosphere, and press setting conditions for the first and second pressing steps were applied identically to all nine cases.

After the second pressing, the toroidal compacts were heat-treated at 720 °C for 30 min under a flowing N_2_–10% H_2_ mixed-gas atmosphere. The heat treatment was performed to relieve residual stresses formed during the compaction process [32], and the heat-treated specimens were used for electromagnetic property evaluation.

After specimen fabrication, the outer diameter, inner diameter, and final height of each case were measured. The final height was measured three times at different positions on each specimen, and the arithmetic mean was calculated. The outer and inner diameters were also measured for the fabricated specimens.

### 2.3. Finite Element Analysis Conditions

Finite element analysis (FEA) was performed using DEFORM-3D to compare the final compact height and relative-density distribution of the toroidal compacts according to the combinations of MoS_2_ and graphite contents [18]. The analysis model was configured to reflect the temperature conditions and pressing sequence of the two-step compaction process used for specimen fabrication. The initial bulk density was set to the powder tap density of 3.34 g/cm^3^, and the initial relative density was set to 0.428 based on the true density of 7.80 g/cm^3^ for the Fe–5.0 wt.%Si powder [5].

The initial powder charge height was calculated by first determining the initial powder volume from the powder charge and tap density used for specimen fabrication and then dividing this volume by the effective cross-sectional area of the toroidal die. The relationships among the initial powder volume, effective cross-sectional area of the toroidal die, and initial powder charge height are given as follows.

(1) $$V_{0}=\frac{m_{0}}{\rho_{\mathrm{tap}}}$$

(2) $$A_{t}=\frac{\pi }{4}(D_{0}^{2}-D_{i}^{2})$$

(3) $$h_{0}=\frac{V_{0}}{A_{t}}=\frac{4m_{0}}{\pi \rho_{\mathrm{tap}}(D_{0}^{2}-D_{i}^{2})}$$

Here, *m*_0_ denotes the powder charge, *ρ_tap_* denotes the tap density of the powder, and *D*_0_ and *D_i_* denote the outer and inner diameters of the toroidal die, respectively. Using a powder charge of 6.0 g and a tap density of 3.34 g/cm^3^ [5], the initial powder volume was calculated to be approximately 1.796 cm^3^. The effective cross-sectional area of the toroidal die was calculated to be approximately 203.1 mm^2^. Based on these values, the initial powder charge height was calculated to be 8.84 mm, and this value was used as the initial height of the FEA model.

The powder compact was defined as a powder-type rigid-plastic deformable body. The yield criterion employed in the present analysis was the default Oyane porous material model implemented in DEFORM-3D [32]. The default model parameters provided by DEFORM-3D were used without user modification. The upper punch, lower punch, and die were defined as rigid bodies [18]. The material hardening behavior was defined using an isotropic hardening formulation with tabulated flow-stress data. The flow stress was defined in DEFORM-3D as a function of effective strain, strain rate, and temperature as follows:

(4) $$\bar{\sigma }=\;\bar{\sigma }(\bar{\varepsilon },\dot{\bar{\varepsilon }},T)$$

where $\bar{\sigma }$ is the flow stress, $\bar{\varepsilon }$ is the effective strain, $\dot{\bar{\varepsilon }}$ is the effective strain rate, and *T* is the temperature. In the present material dataset, a strain rate of 0.1 s^−1^ was used. No separate user-defined relationship between relative density and the matrix flow stress was prescribed; instead, the influence of relative density on the yielding and densification behavior of the powder compact was accounted for through the default Oyane porous material model. Because the powder compact was modeled using a rigid-plastic formulation, elastic deformation was not considered; therefore, the elastic modulus and Poisson’s ratio were not specified as input parameters. The material parameters and constitutive settings used in the FEA are summarized in Table 2.

**Figure 1:**
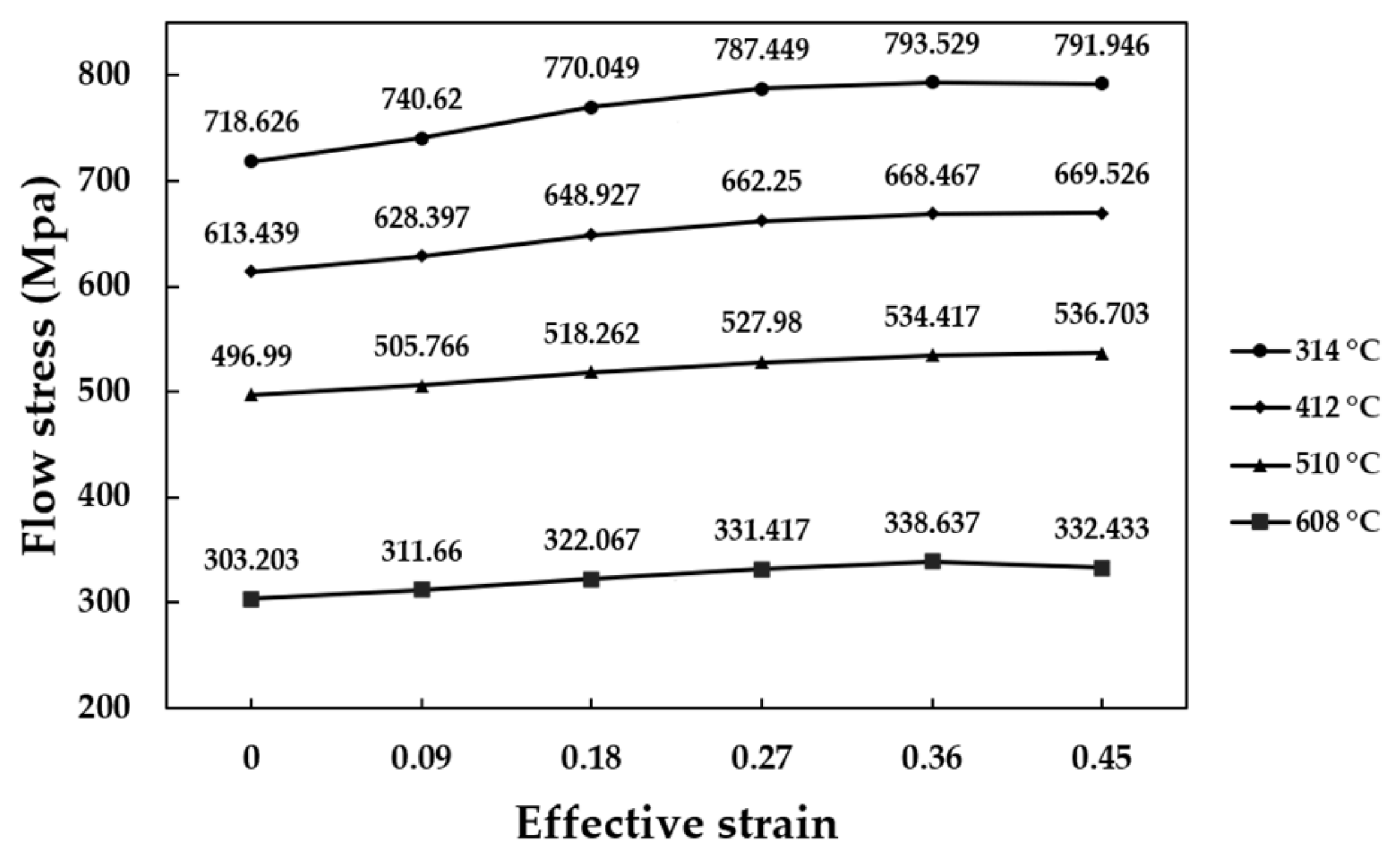
Flow-stress curves of the Fe–5.0 wt.%Si material at the tabulated temperatures of 314, 412, 510, and 608 °C used in the DEFORM-3D material dataset.

The tabulated flow-stress data consisted of six effective-strain input points (0, 0.09, 0.18, 0.27, 0.36, and 0.45) and ten temperature input points (20, 118, 216, 314, 412, 510, 608, 706, 804, and 902 °C), with an effective strain rate of 0.1 s^−1^. Figure 1 presents the flow-stress curves at 314, 412, 510, and 608 °C, which are the tabulated temperature points immediately surrounding the first- and second-pressing temperatures of 350 and 550 °C, respectively.

The powder compact model consisted of tetrahedral elements, with a total of 138,555 elements and 30,368 nodes. The powder–die friction coefficients were assigned using the temperature-dependent friction coefficients obtained for each MoS_2_/graphite composition in the previous study [5]. In the present FEA, the friction coefficient at 350 °C was applied to the first pressing, while the friction coefficient at 550 °C, calculated by extrapolation in the previous study, was applied to the second pressing [5]. Both deformation and heat transfer calculations were enabled, and heat transfer between the powder compact and tooling was considered through the interface heat transfer conditions. Although temperature changes during compaction were calculated, the specific contribution of thermal-expansion deformation associated with friction-induced heating was not separately evaluated. Except for the friction conditions, the initial geometry, powder charge conditions, material properties, and boundary conditions were identical for all cases. The friction coefficients applied to the nine lubricant compositions are summarized in Table 3.

C3 was selected as the reference case for determining the common loads applied to all cases. C3 contained 1.0 wt.% MoS_2_ and 0.3 wt.% graphite and was evaluated as the composition with the best mechanical properties among the nine lubricant composition conditions compared in the previous study [5].

The load-stop conditions for the first and second pressing steps were determined sequentially using the upper-punch load history obtained from the C3 case. First, the C3 case was compressed at 350 °C under displacement control to a final height of 4.0 mm in a single pressing step, and the upper-punch load history generated during this process was extracted. The point corresponding to 50% of the load required to reach a final height of 4.0 mm was then identified, and the corresponding load of 49,947 N was set as the load-stop condition for the first pressing.

Next, the state in which the C3 case had been compressed to 49,947 N at 350 °C was defined as the completion of the first pressing. The pressing temperature was then changed to 550 °C, and the same compact was further compressed to a final height of 4.0 mm. The upper-punch load obtained at this point was 59,566 N, which was set as the load-stop condition for the second pressing. Accordingly, 49,947 and 59,566 N were applied as the common load-stop conditions for the first and second pressing steps, respectively. These loads were not obtained by directly converting the 60 and 120 kgf settings of the specimen fabrication equipment into loads, but were determined separately by FEA using C3 as the reference case.

In the final comparative analyses, the same load-stop conditions were applied to all nine cases from A3 to C8. For all cases, the first pressing at 350 °C was terminated when the upper-punch load reached 49,947 N, and the second pressing at 550 °C was terminated when the load reached 59,566 N. The final compact height was not specified as an input condition in the analysis but was defined as an analysis result determined by the differences in the powder–die friction coefficients applied to each case [5,8,33]. Accordingly, the final compact height and relative-density distribution of each case were compared under the same load-stop conditions [18,33]. The use of C3 as the reference case was limited to establishing the common load-stop conditions. The relative-density distribution was not specified as an input condition for any case but was obtained from the FEA results after applying the same load-stop conditions to all nine cases.

The final compact height and relative-density distribution of each case were extracted from the analysis results. To quantitatively compare the internal density uniformity of the toroidal compacts, the relative-density Max and relative-density Min were obtained for each case, and the difference between these values, relative-density Max–Min, was defined as the internal relative-density range. A smaller relative-density Max–Min indicates a smaller difference between the maximum and minimum relative densities within the specimen and was therefore used to evaluate a more uniform relative-density distribution.

The final compact height obtained for each case from FEA was defined as the FEA-predicted final height and was compared with the experimental average height calculated from three measurements at different positions on the actual toroidal specimen [18]. Finally, the relative-density Max, relative-density Min, and relative-density Max–Min values of each case were compared to evaluate internal relative-density uniformity according to lubricant composition, and the case with the smallest relative-density Max–Min was evaluated as the condition showing the most uniform relative-density distribution in the FEA results.

### 2.4. Electromagnetic Property Evaluation

Inductance, permeability, Q value, core loss, and coercive field were measured to evaluate the electromagnetic properties of the fabricated Fe–5.0 wt.%Si toroidal specimens with nine composition conditions [15]. Inductance, permeability, and Q value were measured using an LCR meter (4284A, Agilent Technologies, Santa Clara, CA, USA), and the same measurement conditions were applied to all cases [15].

For the electromagnetic measurements, the toroidal cores were wound with 20 turns using AWG 28 wire with a conductor diameter of 0.32 mm and an overall diameter of 0.60 mm including insulation. The winding space factor, defined as the ratio of the total copper conductor cross-sectional area to the inner window area of the toroidal core, was approximately 1.30% based on the nominal inner diameter of 12.57 mm.

Inductance, permeability, and Q value were evaluated at 0.5, 1, 2, 5, 10, 20, 50, and 100 kHz [15]. The values directly obtained from the instrument under the specified measurement conditions were used. For permeability measurements, the actual dimensions of each specimen were entered into the instrument to account for geometric differences among the cases [15].

Core loss was measured using a B–H analyzer (SY-8216, IWATSU Electric Co., Ltd., Tokyo, Japan) [34]. The magnetic flux density was fixed at 300 mT, and the measurement frequencies were set to 1000 and 1500 Hz. At each measurement condition, the volumetric core loss, *P*_cv_, mass-specific core loss, *P*_cm_, and coercive field, *H*_c_, directly obtained from the B–H analyzer were used [34]. *P*_cv_ was expressed in kW/m^3^, *P*_cm_ in W/kg, and *H*_c_ in A/m. The same magnetic flux density and frequency conditions were applied to all cases to compare core loss and coercive field according to the combinations of MoS_2_ and graphite contents [24,34,35].

The frequency-dependent changes in inductance, permeability, and Q value, together with *P*_cv_, *P*_cm_, and *H*_c_ measured at 1000 and 1500 Hz under 300 mT, were compared with the final compact height, relative-density distribution, and microstructural analysis results obtained for each case from FEA [15]. These comparisons were used to examine the relationships among compaction state, microstructure, and electromagnetic properties according to the combinations of MoS_2_ and graphite contents [15,24,35].

### 2.5. Microstructural and Elemental Distribution Analysis

Cross-sectional observations were performed for all nine cases using scanning electron microscopy (SEM; JEOL Ltd., Tokyo, Japan) to compare microstructural differences according to the MoS_2_/graphite lubricant compositions [5,15,27]. The particle morphology and deformation state, interparticle contact conditions, pore morphology and distribution, and final densification state were observed in the cross sections of the Fe–5.0 wt.%Si toroidal specimens [5,15,16,27]. The cross-sectional microstructures of each case were also compared to analyze microstructural differences according to the combinations of MoS_2_ and graphite contents [5,15]. For the SEM analysis, only clearly resolved void regions between adjacent particles were described as pores. Ambiguous dark linear features that could not be clearly identified as voids from the SEM morphology alone were not classified as pores or cracks. Accordingly, the SEM comparison focused on clearly identifiable pores and the overall interparticle contact state.

Digital image analysis was additionally performed using Fiji (ImageJ, version 1.54p) to quantify the two-dimensional pore area fraction in the SEM micrographs. The SEM images acquired at ×1000 magnification were analyzed using the same region-of-interest selection and thresholding procedure for all cases. Only clearly resolved void regions between adjacent particles were classified as pores, whereas ambiguous dark linear features, particle boundaries, and polishing artifacts were excluded. The two-dimensional pore area fraction was calculated as the ratio of the segmented pore area to the total analyzed area.

Transmission electron microscopy–energy dispersive X-ray spectroscopy (TEM–EDS) was performed for five cases, A3, B3, C3, C5, and C8, to analyze the local microstructure and elemental distribution at the interparticle interfaces [7,15,36]. The TEM–EDS cases were selected using C3 as the reference case because it showed the best mechanical properties in the previous study [5]. Through this comparison, the interparticle interface characteristics were evaluated according to the individual changes in MoS_2_ and graphite contents [5,15,24]. For each case, two different specimens were selected, and one TEM lamella was prepared from each specimen, resulting in a total of two lamellae per case. The interface microstructure and elemental distribution observed in the two lamellae from the same case were compared together.

Thin specimens for TEM–EDS analysis were prepared using a focused ion beam (FIB) system (Helios 5 CX, Thermo Fisher Scientific, Waltham, MA, USA) [37]. For each case, the same FIB processing location was selected to include the interfacial region between Fe–Si particles. The selected region was selectively extracted using the FIB and processed into an electron-transparent thin specimen suitable for TEM analysis [37]. However, regions damaged by FIB processing were excluded from the subsequent interfacial region thickness measurements [37].

A field-emission transmission electron microscope (FE-TEM; Talos F200i, Thermo Fisher Scientific, Waltham, MA, USA) was used for TEM–EDS analysis [7,15,36]. The analysis region was centered on the interface between Fe–Si powder particles at the same location for each case, and the interparticle interface microstructure, local pores, and interface morphology were observed [7,15,36]. In this study, the region containing interfacial components between adjacent Fe–Si particles was defined as the interfacial region, and regions in which lubricant- and additive-derived components were locally distributed were also included in the interfacial region. In contrast, clearly identified pores and regions damaged by FIB processing were excluded from the interfacial region thickness measurements [37]. The interfacial region thickness was measured at a total of 10 points in each TEM lamella in the direction perpendicular to the interface, and the minimum, maximum, and mean values were calculated for each lamella to quantitatively compare the interfacial region thickness characteristics among the cases.

EDS mapping was also performed to analyze the local distributions of Fe, Si, O, P, C, Mo, and S [7,15,17,36]. The Fe–Si particles and interfacial regions were distinguished based on the distributions of Fe and Si [7,15,36]. The spatial distributions of P and O were used to identify the distribution of P–O-containing interfacial components associated with H_3_PO_4_ treatment [6,15,27], while the distributions of Mo and S were used to compare the distribution of Mo- and S-containing interfacial components associated with MoS_2_ addition [5,15,24,31]. C was used to compare the spatial distribution of C-containing interfacial regions associated with carbon-based additives, including graphite and polyimide [5,15,27,29]. Regions in which Si and O appeared together at some interfaces were analyzed as local interfacial regions in which Si and O were spatially overlapped [7,27,36]. The EDS mapping results were compared based primarily on the locations and spatial distributions of the elements within the particles and interfaces rather than on their signal intensities.

The SEM and TEM–EDS results were compared with the relative-density distributions obtained from FEA and the electromagnetic property results [7,15,36]. The interfacial region thickness and spatial elemental distributions obtained from TEM–EDS were used as analysis results representing local interface characteristics [7,15,36]. These results were not interpreted as a single cause of the electromagnetic properties but were evaluated together with the relative-density Max, relative-density Min, and relative-density Max–Min obtained from FEA, the SEM observations, and the electromagnetic properties.

## 3. Simulation and Electromagnetic Property Results

### 3.1. FEA Model Geometry and Comparison of Final Compact Heights

The same analysis model geometry was applied to all cases to compare the final compaction characteristics of the toroidal compacts according to the combinations of MoS_2_ and graphite contents. The same first- and second-pressing load-stop conditions were applied to all cases, while different powder–die friction coefficients corresponding to each lubricant composition were used [5]. Accordingly, the final compact heights were compared according to the differences in friction coefficients among the cases under the same load-stop conditions [5,18,33].

**Figure 2:**
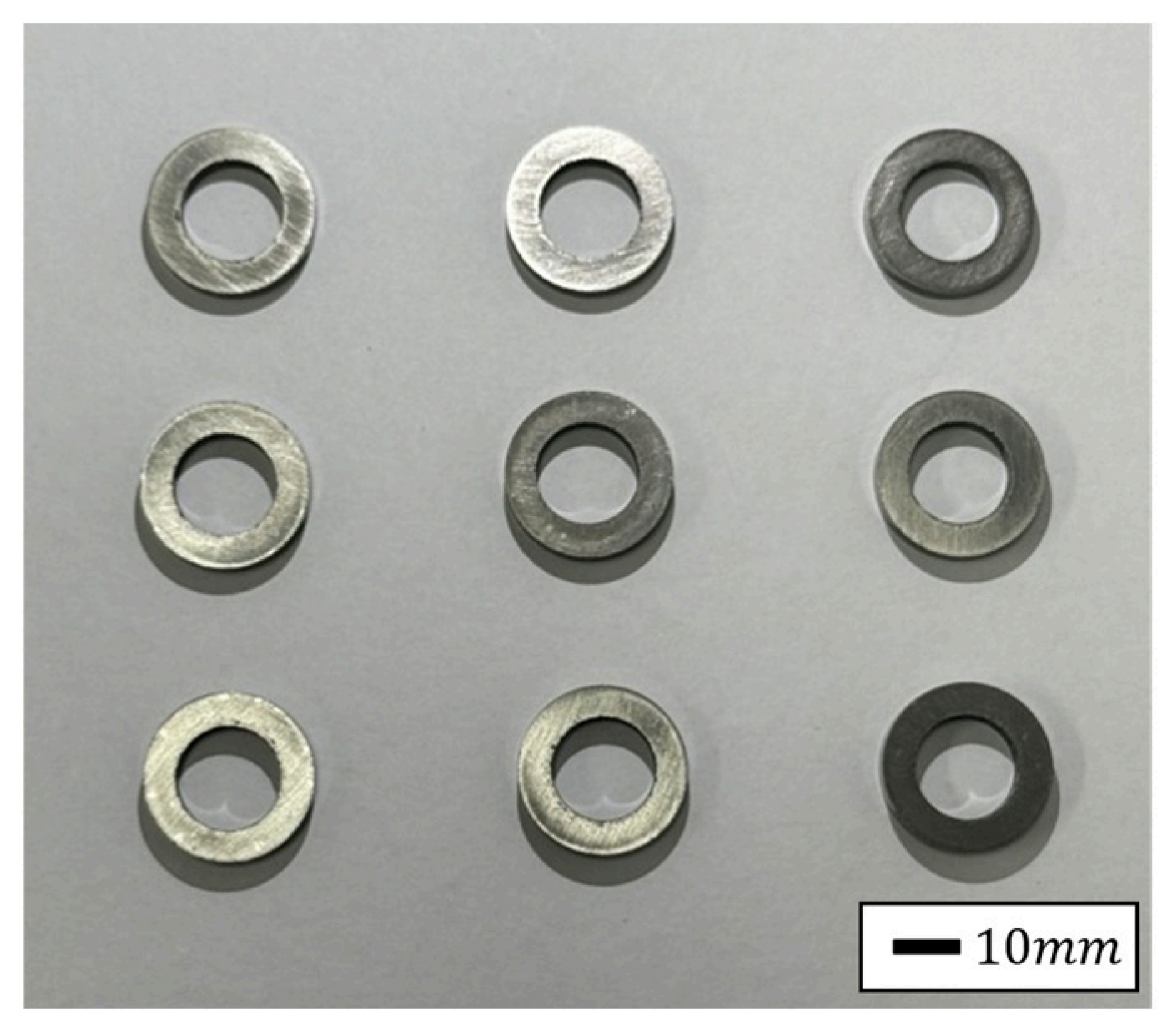
Fe–5.0 wt.%Si toroidal specimens fabricated with nine MoS_2_/graphite lubricant compositions: A3, A5, and A8 in the top row; B3, B5, and B8 in the middle row; and C3, C5, and C8 in the bottom row. Scale bar: 10 mm.

Figure 2 shows the Fe–5.0 wt.%Si toroidal specimens fabricated with the nine MoS_2_/graphite lubricant composition conditions listed in Table 1. The specimens were arranged in a 3 × 3 configuration according to lubricant composition. A3, A5, and A8 were placed in the top row; B3, B5, and B8 in the middle row; and C3, C5, and C8 in the bottom row. Within each row, the graphite content increased from left to right in the order of 0.3, 0.5, and 0.8 wt.%. All specimens retained their overall toroidal shape after the same 2P1A compaction and heat-treatment process, and no distinct shape collapse or macroscopic fracture was observed by visual inspection.

After specimen fabrication, the final height of each case was measured three times at different positions, and the arithmetic mean was used as the experimental average height. The final heights obtained from FEA were compared with the experimental average heights of the fabricated specimens in Table 4. The relative error between the FEA-predicted and experimental values was calculated based on the experimental average height of the fabricated specimen as follows.

(5) $$E_{h}=\frac{\left|h_{\mathrm{FEA}}-h_{\exp}\right|}{h_{\exp}}\times 100$$

Here, E_h_ denotes the relative error in the final height, h_FEA_ denotes the final compact height predicted by FEA, and h_exp_ denotes the experimental average height of the fabricated specimen.

The FEA-predicted final compact heights ranged from 3.97 to 4.03 mm, while the average heights of the fabricated specimens ranged from 3.99 to 4.04 mm. For A5, A8, and C5, the FEA-predicted heights were identical to the experimental average heights. For A3, B5, and C3, the difference between the two values was 0.01 mm, corresponding to a relative error of 0.25%. The height differences for B3 and C8 were 0.03 and 0.04 mm, respectively, with relative errors of 0.75% and 0.99%, respectively.

For all cases, the relative error between the FEA-predicted height and the experimental average height was less than 2%. Excluding C3, which was used to establish the common load-stop conditions, the mean relative error for the remaining eight cases was approximately 0.50%, and the mean absolute height difference was approximately 0.020 mm. Therefore, for the remaining cases compared independently of the reference case, the difference between the FEA-predicted height and the experimental average height was within 2%, indicating that the FEA results were reliable.

### 3.2. Relative-Density Distribution and Internal Density Uniformity

**Figure 3:**
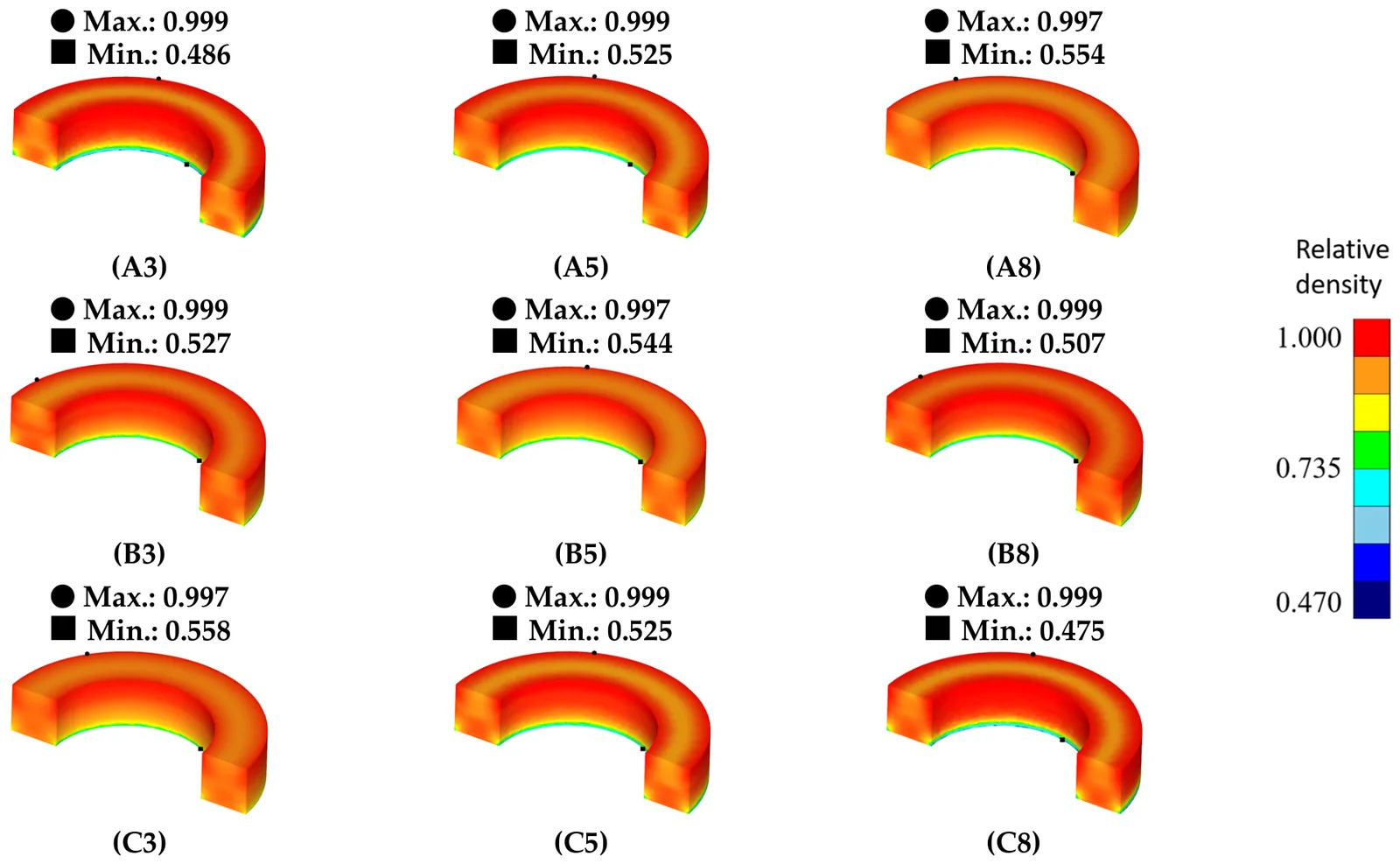
Final relative-density distributions obtained from FEA for the Fe–5.0 wt.%Si toroidal compacts with nine MoS_2_/graphite lubricant compositions, including the maximum and minimum relative densities for each case.

The internal relative-density distributions of the toroidal compacts were compared according to the combinations of MoS_2_ and graphite contents. Each case was analyzed at the final compaction state corresponding to the second pressing load-stop condition of 59,566 N, and internal density uniformity was evaluated using the relative-density Max, relative-density Min, and relative-density Max–Min. Figure 3 shows the final relative-density distributions of the nine cases together with the relative-density Max and relative-density Min obtained for each compact. The same legend range was applied to all analysis results to allow direct comparison of the relative-density levels and distributions among the cases.

In all cases, most regions of the compacts showed relatively high relative density, whereas local low-density regions were formed near the die-contact boundary at the inner lower region. The relative-density Max was mainly observed in regions adjacent to the upper punch, whereas the relative-density Min was mainly identified near the inner lower region and die-contact area of the toroidal compact. Differences in relative density were formed depending on the position within the compact due to compression by the upper punch and powder–die friction [8,18,33], and differences in the applied friction coefficients among the cases also resulted in differences in the low-density regions and relative-density Min [5,8,33].

The relative-density Max values of the cases ranged from 0.997 to 0.999, showing only small differences according to lubricant composition. The relative-density Max values of A3, A5, B3, B8, C5, and C8 were 0.999, whereas those of A8, B5, and C3 were 0.997.

In contrast, the relative-density Min values ranged from 0.475 to 0.558, showing greater differences among the cases than the relative-density Max values. The relative-density Min values of A3, A5, and A8 were 0.486, 0.525, and 0.554, respectively, while those of B3, B5, and B8 were 0.527, 0.544, and 0.507, respectively. The relative-density Min values of C3, C5, and C8 were 0.558, 0.525, and 0.475, respectively. Among these cases, C3 showed the highest relative-density Min of 0.558, whereas C8 showed the lowest value of 0.475. Therefore, the differences in internal density uniformity among the cases were more evident in the variation in relative-density Min than in relative-density Max.

**Figure 4:**
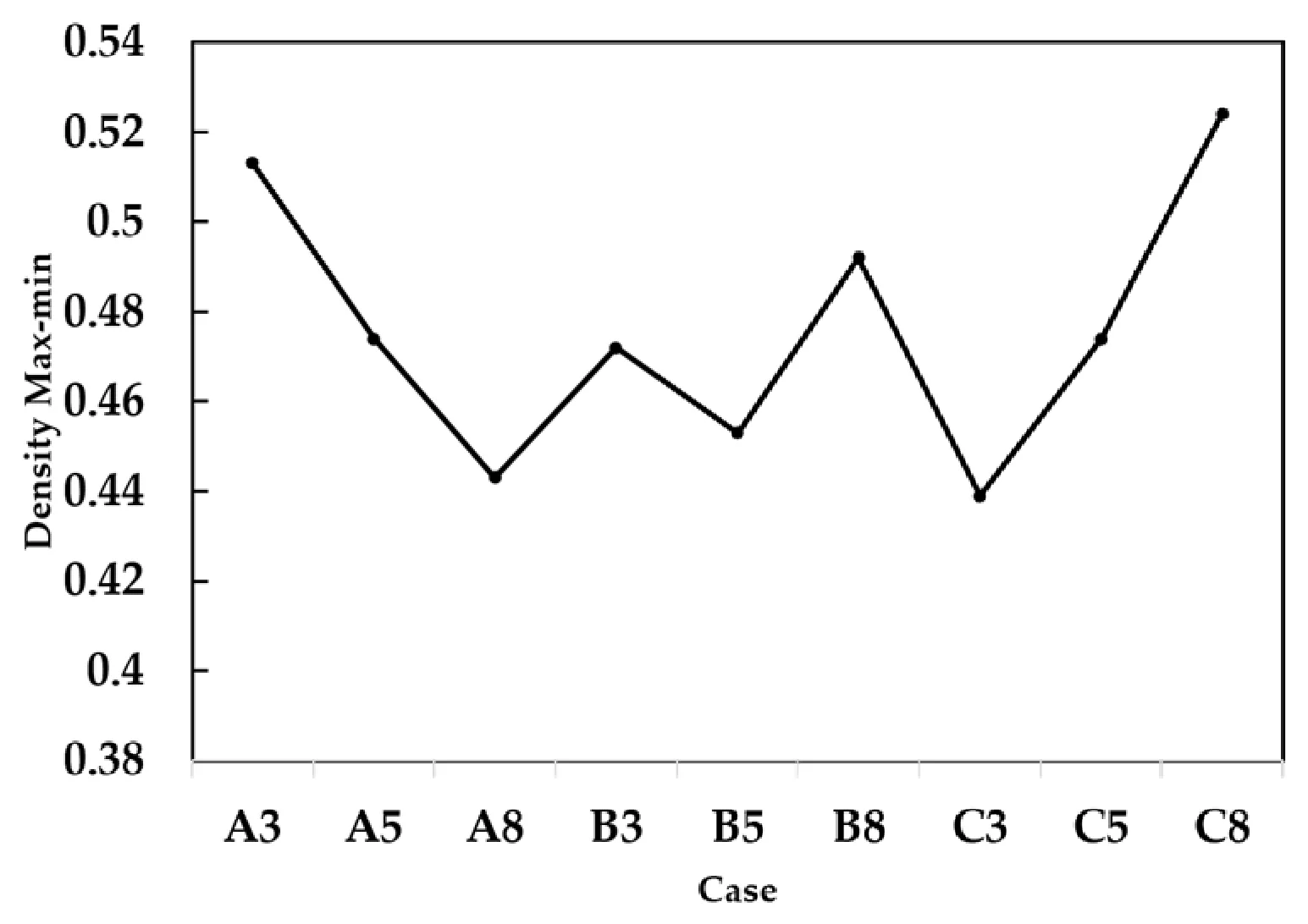
Differences between the maximum and minimum relative densities obtained from FEA for the Fe–5.0 wt.%Si toroidal compacts with nine MoS_2_/graphite lubricant compositions.

To quantitatively compare the internal density uniformity of each case, the relative-density Max–Min, defined as the difference between relative-density Max and relative-density Min, was analyzed. Figure 4 shows the relative-density Max–Min values of the nine cases. A smaller value indicates a smaller difference between the maximum and minimum relative densities within the compact and was therefore used to evaluate a more uniform internal relative-density distribution.

The relative-density Max–Min values of A3, A5, and A8 were 0.513, 0.474, and 0.443, respectively. Those of B3, B5, and B8 were 0.472, 0.453, and 0.492, respectively, while those of C3, C5, and C8 were 0.439, 0.474, and 0.524, respectively. Among the nine cases, C3 showed the smallest relative-density Max–Min of 0.439, followed by A8 with 0.443.

The relative-density Max of C3 was 0.997, similar to those of the other cases, whereas its relative-density Min was maintained at 0.558, the highest among all cases. Therefore, the relative-density Max–Min of C3 was more directly associated with the relative-density Min than with the relative-density Max. In particular, the relative density in the local low-density region at the inner lower region was maintained at a higher level than in the other cases, and the C3 condition showed a favorable result in terms of improving internal density uniformity.

In contrast, C8 showed a relative-density Max of 0.999 and a relative-density Min of 0.475, resulting in the largest relative-density Max–Min of 0.524. A3 also showed a low relative-density Min of 0.486, resulting in a large relative-density Max–Min of 0.513. In C8 and A3, the relative-density Min values were 0.475 and 0.486, respectively, and accordingly, their relative-density Max–Min values were also large at 0.524 and 0.513, respectively.

When the effect of graphite content was compared at the same MoS_2_ content, the relative-density Max–Min in the A series decreased from 0.513 for A3 to 0.443 for A8 as the graphite content increased from 0.3 to 0.8 wt.%. In the B series, B5 showed the smallest value of 0.453, whereas the value increased again to 0.492 for B8 containing 0.8 wt.% graphite. In the C series, C3, with the lowest graphite content, showed the most uniform relative-density distribution with a relative-density Max–Min of 0.439, while the relative-density Max–Min values increased to 0.474 and 0.524 for C5 and C8, respectively, as the graphite content increased. Therefore, the change in internal density uniformity with increasing graphite content showed different trends depending on the MoS_2_ content.

In particular, C3 was the composition that showed the best mechanical properties in the previous study and, in the present FEA, also showed the smallest relative-density Max–Min of 0.439 and the highest relative-density Min of 0.558 among the nine cases. Under the C3-derived common load-stop conditions used in this study, the C3 composition containing 1.0 wt.% MoS_2_ and 0.3 wt.% graphite maintained a relatively high relative density in the local low-density region and showed the smallest relative-density Max–Min and the highest relative-density Min among the nine cases.

### 3.3. Electromagnetic Property Results According to Lubricant Composition

Inductance, permeability, Q value, volumetric core loss, P_cv_, mass-specific core loss, P_cm_, and coercive field, H_c_, were measured to evaluate the electromagnetic properties of the fabricated Fe–5.0 wt.%Si toroidal cores with nine composition conditions [15,34]. The frequency-dependent measurement results for inductance, permeability, and Q value are presented separately in Supplementary Materials S1. In the main text, the P_cv_, P_cm_, and H_c_ results were mainly analyzed to clearly compare the effects of the MoS_2_/graphite content combinations on magnetic loss characteristics. For each case, three different toroidal cores were measured under the same conditions, and the arithmetic mean of the values obtained from the three cores was used as the representative value for that case.

**Figure 5:**
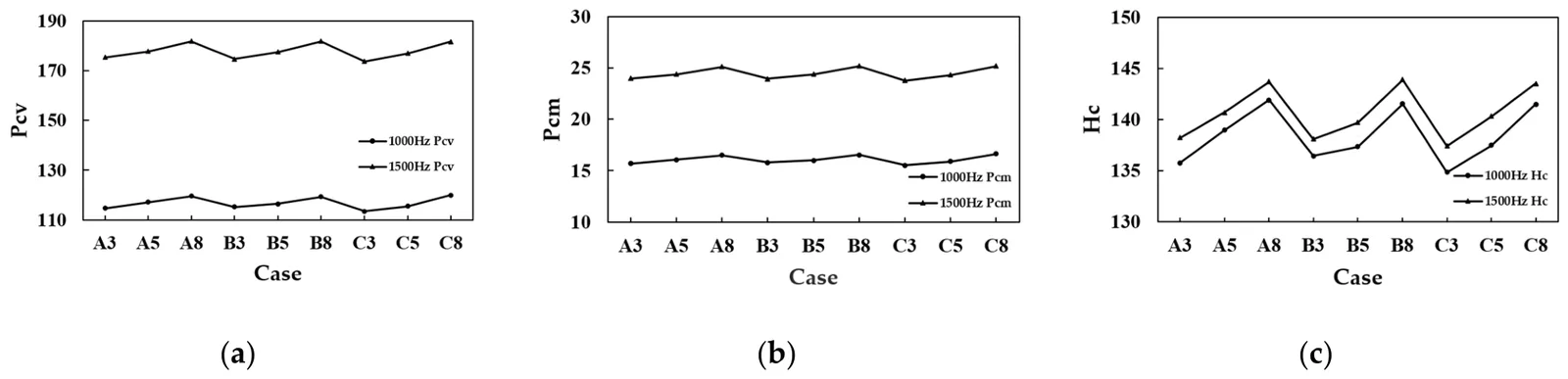
Core-loss and coercivity characteristics of the Fe–5.0 wt.%Si toroidal cores with nine MoS_2_/graphite lubricant compositions at 1000 and 1500 Hz under a maximum magnetic flux density of 300 mT: (**a**) volumetric core loss, P_cv_; (**b**) mass-specific core loss, P_cm_; and (**c**) coercive field, H_c_. Each value represents the arithmetic mean of three separate toroidal cores (*n* = 3).

Core loss and coercive field were measured at frequencies of 1000 and 1500 Hz under a maximum magnetic flux density of 300 mT. Figure 5 compares P_cv_, P_cm_, and H_c_ at 1000 and 1500 Hz using the mean values measured from three toroidal cores for each case. P_cv_ and P_cm_ represent the core loss per unit volume and per unit mass, respectively, and lower values are favorable in terms of magnetic loss [1,2,35]. In addition, a lower H_c_ indicates a smaller magnetic field required to reverse the magnetization direction and is therefore favorable in terms of coercivity [2,35].

At 1000 Hz and 300 mT, the measured P_cv_ ranged from 113.50 to 119.97 kW/m^3^. The mean P_cv_ values of A3, A5, and A8 were 114.63, 117.17, and 119.53 kW/m^3^, respectively, while those of B3, B5, and B8 were 115.23, 116.40, and 119.33 kW/m^3^, respectively. C3, C5, and C8 showed values of 113.50, 115.53, and 119.97 kW/m^3^, respectively. Among these cases, C3 showed the lowest value of 113.50 kW/m^3^, whereas C8 showed the highest value of 119.97 kW/m^3^.

As the measurement frequency increased to 1500 Hz, P_cv_ increased in all cases [24,25,35]. The mean P_cv_ values of A3, A5, and A8 were 175.33, 177.87, and 181.90 kW/m^3^, respectively, while those of B3, B5, and B8 were 174.73, 177.60, and 181.90 kW/m^3^, respectively. C3, C5, and C8 showed values of 173.70, 176.97, and 181.70 kW/m^3^, respectively. At 1500 Hz, C3 also showed the lowest P_cv_ of 173.70 kW/m^3^. Therefore, C3 showed the lowest volumetric core loss at both 1000 and 1500 Hz and showed the best result in terms of magnetic loss per unit volume.

At 1000 Hz, the mean P_cm_ values of A3, A5, and A8 were 15.68, 16.06, and 16.51 W/kg, respectively, while those of B3, B5, and B8 were 15.80, 16.00, and 16.53 W/kg, respectively. C3, C5, and C8 showed values of 15.54, 15.88, and 16.64 W/kg, respectively. Among the nine cases, C3 showed the lowest P_cm_ of 15.54 W/kg, followed by A3 with 15.68 W/kg.

At 1500 Hz, the mean P_cm_ values of A3, A5, and A8 were 23.97, 24.38, and 25.12 W/kg, respectively, while those of B3, B5, and B8 were 23.96, 24.41, and 25.20 W/kg, respectively. C3, C5, and C8 showed values of 23.78, 24.32, and 25.19 W/kg, respectively. At 1500 Hz, C3 also showed the lowest P_cm_ of 23.78 W/kg. Therefore, C3 showed the lowest mass-specific core loss at both 1000 and 1500 Hz and showed the best results for both P_cv_ and P_cm_ compared with the other cases.

At 1000 Hz, the mean H_c_ values ranged from 134.83 to 141.91 A/m. The H_c_ values of A3, A5, and A8 were 135.76, 138.97, and 141.91 A/m, respectively, while those of B3, B5, and B8 were 136.43, 137.35, and 141.53 A/m, respectively. C3, C5, and C8 showed values of 134.83, 137.47, and 141.50 A/m, respectively. C3 showed the lowest H_c_ of 134.83 A/m.

At 1500 Hz, the mean H_c_ values of A3, A5, and A8 were 138.22, 140.68, and 143.69 A/m, respectively, while those of B3, B5, and B8 were 138.07, 139.71, and 143.89 A/m, respectively. C3, C5, and C8 showed values of 137.40, 140.30, and 143.52 A/m, respectively. At 1500 Hz, C3 also showed the lowest coercive field of 137.40 A/m. Therefore, C3 showed the lowest H_c_ at both frequencies, indicating a relatively smaller magnetic field required for magnetization reversal and a favorable result in terms of hysteresis loss [2,35].

When the effect of graphite content was compared at the same MoS_2_ content, P_cv_, P_cm_, and H_c_ increased with graphite content in all three series (A, B, and C) at both 1000 and 1500 Hz. In particular, A8, B8, and C8, each containing 0.8 wt.% graphite, exhibited higher core loss and coercive field than the corresponding cases with lower graphite contents. This behavior may be associated with changes in interparticle electrical insulation and magnetic flux continuity caused by increasing the graphite content. Because graphite is electrically conductive, excessive graphite addition may promote local conductive paths between particles, potentially reducing interparticle insulation resistance and increasing the eddy-current loss contribution. In addition, an increased proportion of nonmagnetic graphite at the interparticle interfaces may interrupt magnetic flux continuity and contribute to higher coercive fields [15]. These mechanisms are considered possible contributions to the observed magnetic loss characteristics, as the individual loss components and interparticle electrical resistance were not independently measured.

In contrast, when the effect of MoS_2_ content was compared at a fixed graphite content of 0.3 wt.%, C3 showed the lowest P_cv_, P_cm_, and H_c_ among A3, B3, and C3 at both 1000 and 1500 Hz. That is, under the condition of 0.3 wt.% graphite, C3 containing 1.0 wt.% MoS_2_ showed the lowest core loss and coercive field. These results show that the magnetic loss characteristics of the toroidal cores varied depending on the combinations of MoS_2_ and graphite contents [15,21,24].

Under the C3-derived common load-stop conditions, C3 showed the smallest relative-density Max–Min of 0.439 and the highest relative-density Min of 0.558 in the FEA. Independently, the electromagnetic measurements of the fabricated toroidal cores showed that C3 had the lowest P_cv_, P_cm_, and H_c_ at both 1000 and 1500 Hz among the nine investigated compositions. Thus, the FEA-based relative-density characteristics under the specified common load-stop conditions and the experimentally measured magnetic-loss characteristics were evaluated as separate results and were subsequently considered together.

## 4. Microstructural Analysis and Discussion

### 4.1. SEM Microstructural Analysis Results

**Figure 6:**
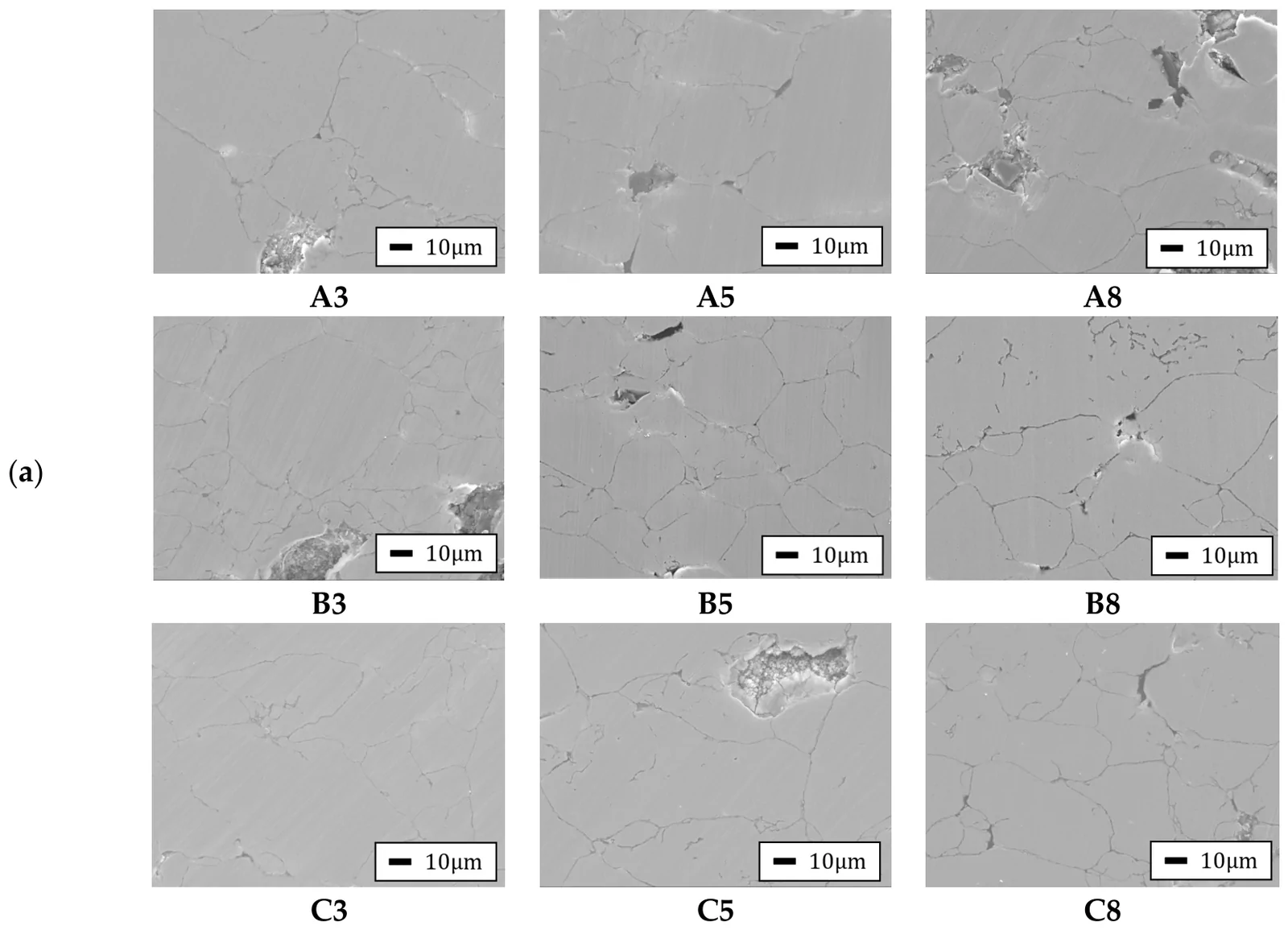

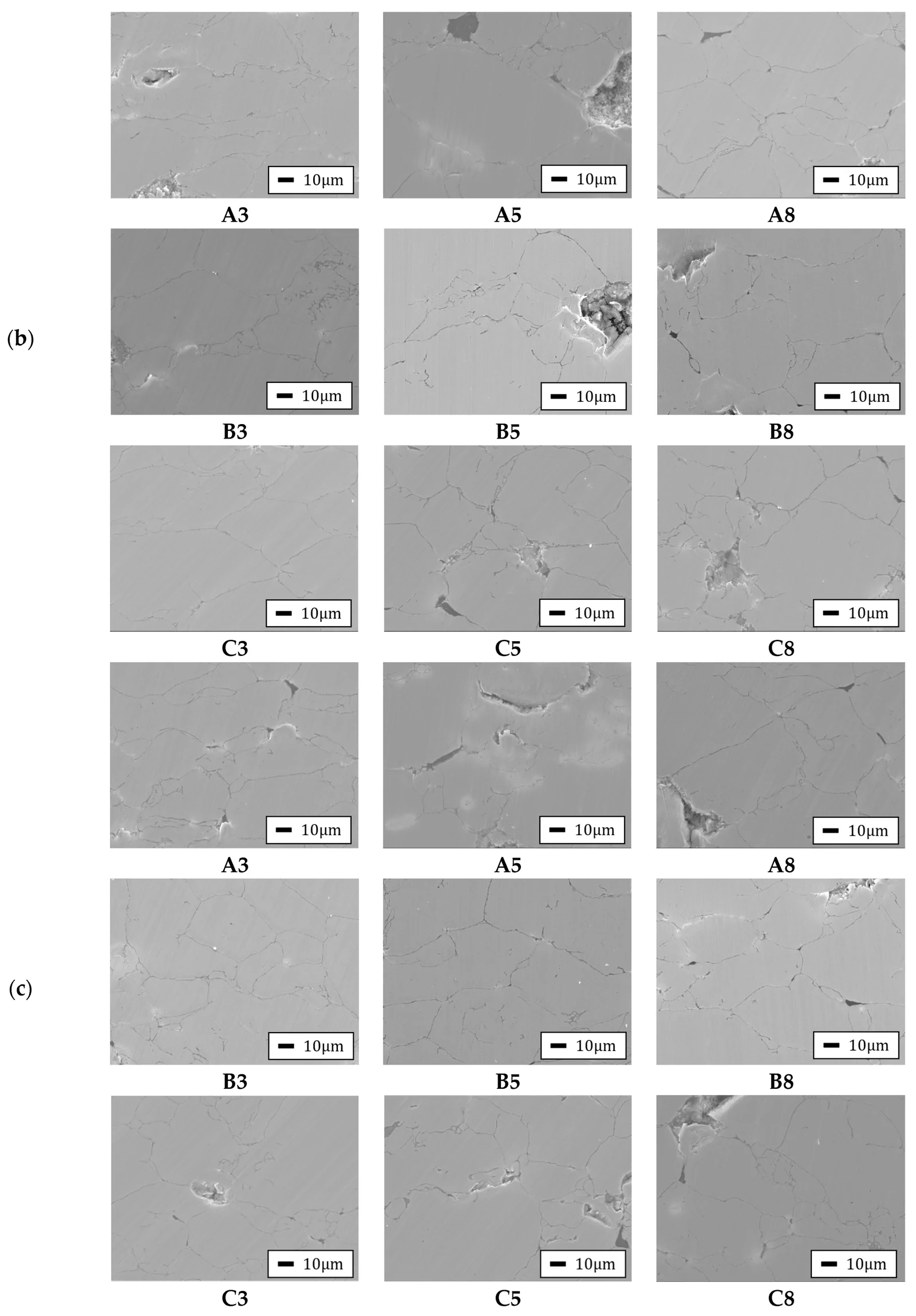
SEM micrographs of the Fe–5.0 wt.%Si toroidal compacts with nine MoS_2_/graphite lubricant compositions observed at ×1000 magnification at different positions along the compact height: (**a**) Top, (**b**) Middle, and (**c**) Bottom. The cases are arranged as A3, A5, A8, B3, B5, B8, C3, C5, and C8 in each panel. Scale bars: 10 μm.

SEM observations were performed for all nine cases to compare the final densification states and microstructural differences in the Fe–5.0 wt.%Si toroidal compacts according to the combinations of MoS_2_ and graphite contents [5,15]. To examine microstructural variations along the specimen height, the Top, Middle, and Bottom positions of each specimen were selected, and all images were acquired at the same magnification of ×1000. Figure 6 compares the SEM microstructures of the nine cases at different positions. Figure 6a, Figure 6b, and Figure 6c show the images observed at the Top, Middle, and Bottom positions, respectively. At each position, the cases were arranged in the same order of A3, A5, A8, B3, B5, B8, C3, C5, and C8 to compare microstructural differences according to lubricant composition.

Although the same compaction and heat-treatment conditions were applied to all cases, the SEM images showed differences in interparticle contact conditions and in pore size and distribution depending on the lubricant composition [5,15,21,24]. Some microstructural differences were also observed among the Top, Middle, and Bottom positions within the same case, indicating differences in the final densification state along the specimen height [18,33].

At the Top position in Figure 6a, clearly identifiable local pores and differences in interparticle contact state were observed in several cases, including A3, A8, B3, and C5. Small local pores were also observed in A5, B5, and B8. In contrast, C3 showed a relatively continuous interparticle contact state compared with the other cases.

Differences among the cases were also observed in the SEM images at the Middle position in Figure 6b. Local pores were observed in A3 and A5, with a relatively large local pore observed in A5. Local pores were also observed in B5 and C8, while C5 showed locally reduced interparticle contact. In contrast, C3 maintained a relatively continuous interparticle contact state at the Middle position and showed interparticle contact conditions and a final densification morphology similar to those observed at the Top position.

At the Bottom position in Figure 6c, more pronounced local pores and differences in interparticle contact state than those at the Top and Middle positions were observed in some cases. Relatively large local pores were identified in A5 and A8, and local pores were also observed in C5 and C8. B3 and B5 showed relatively uniform microstructures, while C3 also contained some small pores at the Bottom position, but its overall interparticle contact state remained relatively continuous.

When the Top, Middle, and Bottom positions were compared together, some cases showed differences in pore size and interparticle contact conditions depending on the position. In contrast, C3 maintained a relatively consistent interparticle contact state at all three positions and showed relatively fewer large local pores. Accordingly, C3 exhibited the most uniform final densification state along the height direction among the nine cases. To quantitatively evaluate the pores observed in the SEM micrographs, the two-dimensional pore area fractions at the Top, Middle, and Bottom positions were determined for all nine cases and are summarized in Table 5. Digital image analysis showed that the two-dimensional pore area fractions of C3 were 1.856%, 1.945%, and 2.827% at the Top, Middle, and Bottom positions, respectively. C3 exhibited the lowest pore area fraction among the nine cases at both the Top and Middle positions and maintained a relatively low pore area fraction at the Bottom position. These quantitative results were consistent with the SEM observations showing relatively continuous interparticle contact and limited formation of large local pores in C3.

These SEM observations showed a trend consistent with the relative-density distribution obtained from FEA. C3 showed the highest relative-density Min of 0.558 and the smallest relative-density Max–Min of 0.439 among the nine cases. In other words, the internal relative-density uniformity of C3 identified by FEA and the interparticle contact and pore distribution observed by SEM along the height direction showed similar trends.

C3 also showed the lowest P_cv_, P_cm_, and H_c_ at both 1000 and 1500 Hz in the electromagnetic property evaluation. Therefore, in C3, the internal relative-density uniformity identified by FEA, the microstructural uniformity along the height direction observed by SEM, and the low core loss and coercive field were observed together.

### 4.2. TEM–EDS Analysis Results

TEM–EDS analysis was performed for A3, B3, C3, C5, and C8 to compare the interparticle interface microstructure and elemental distribution according to the combinations of MoS_2_ and graphite contents [7,15,36,38,39,40]. For each case, one TEM lamella was prepared from each of two different specimens, and the interparticle interface microstructure and interfacial region thickness were compared.

**Figure 7:**
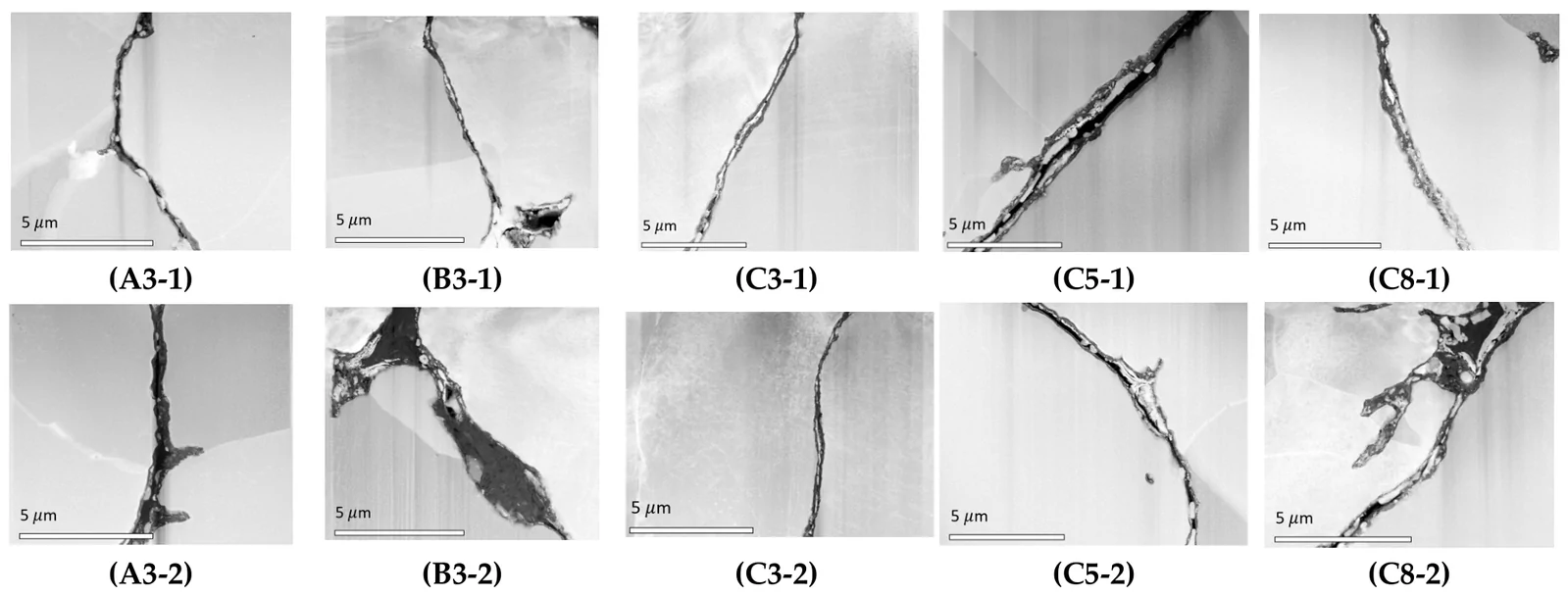
TEM images of the interparticle interface regions in the A3, B3, C3, C5, and C8 cases. A3-1, B3-1, C3-1, C5-1, and C8-1 are shown in the top row, and A3-2, B3-2, C3-2, C5-2, and C8-2 are shown in the bottom row. The suffixes -1 and -2 indicate TEM lamellae prepared from two different specimens of the same case. Scale bars: 5 μm.

Figure 7 shows the TEM images observed in the two lamellae of A3, B3, C3, C5, and C8. The two lamellae of each case are presented together to compare differences in interparticle interface morphology and interfacial region thickness within the same case. In A3 and B3, relatively large differences in the morphology and thickness of the interfacial regions were observed between the two lamellae, and locally expanded interfacial regions were also observed in C5 and C8 depending on the lamella. In contrast, relatively thin interfacial regions were observed in both lamellae prepared from two different C3 specimens.

To quantitatively compare the differences in interface structure observed in the TEM images, the interfacial region thickness was measured at 10 points in each lamella, and the minimum, maximum, and mean values obtained for each lamella are summarized in Table 6 [38,39]. The mean interfacial region thicknesses of A3-1 and A3-2 were 223.7 and 678.5 nm, respectively, while those of B3-1 and B3-2 were 474.9 and 1426.1 nm, respectively. In particular, a locally expanded interfacial region with a maximum thickness of 2400 nm was measured in B3-2.

The mean interfacial region thicknesses of C3-1 and C3-2 were 99.9 and 89.9 nm, respectively, with measurement ranges of 43–181 nm and 51–152 nm, respectively. The mean thicknesses of the two lamellae were similar, and all measured interfacial regions were distributed within a range below 200 nm. Therefore, in the present TEM observations, C3 showed thin and similar interfacial regions in both specimens.

The mean interfacial region thicknesses of C5-1 and C5-2 were 351.7 and 163.5 nm, respectively, while those of C8-1 and C8-2 were 339.8 and 671.8 nm, respectively. In particular, a locally expanded interfacial region with a maximum thickness of 1290 nm was identified in C8-2. In the A3–B3–C3 comparison, no consistent increase or decrease in interfacial region thickness was observed with increasing MoS_2_ content, and no monotonic change in thickness with increasing graphite content was identified in the C3–C5–C8 comparison. In other words, the local interfacial region thickness did not simply correspond to changes in the bulk lubricant content, and differences in interface morphology and thickness were observed among lamellae even within the same composition. In addition, A3, B3, C5, and C8 showed larger differences in mean thickness between the two lamellae than C3.

**Figure 8:**
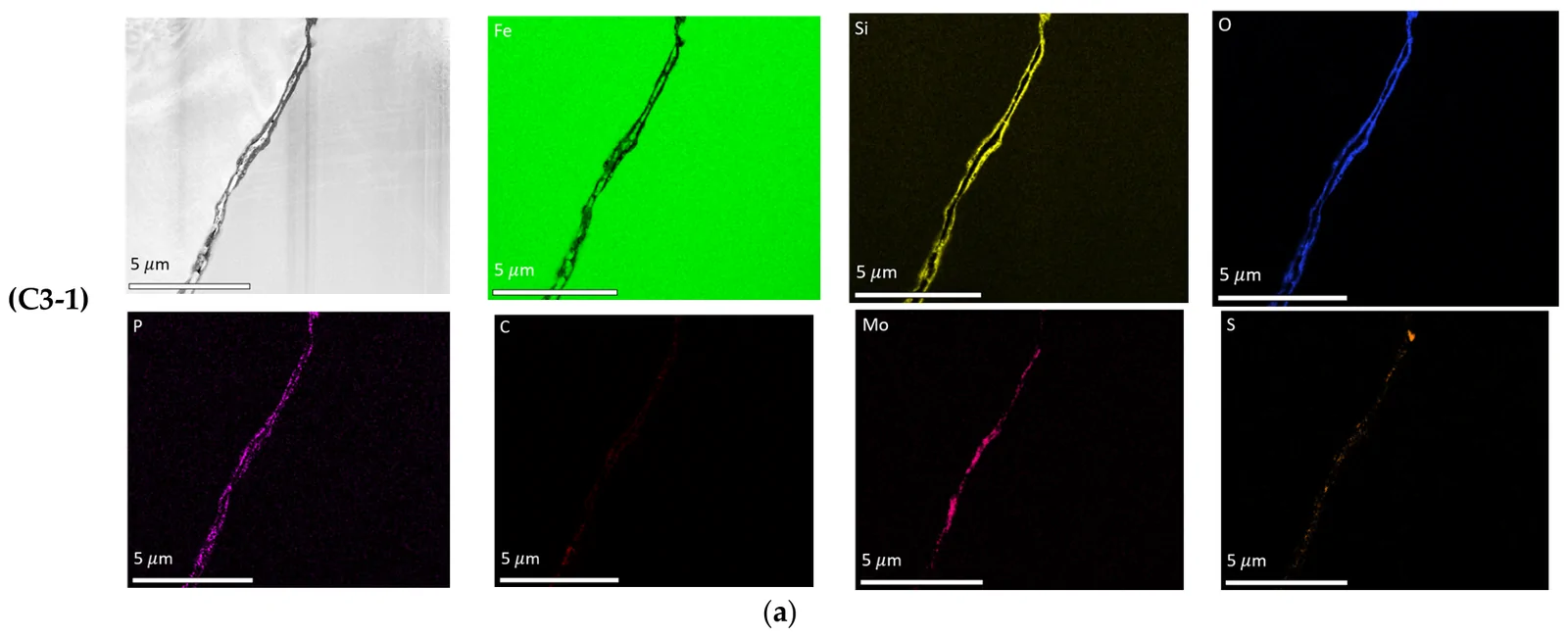

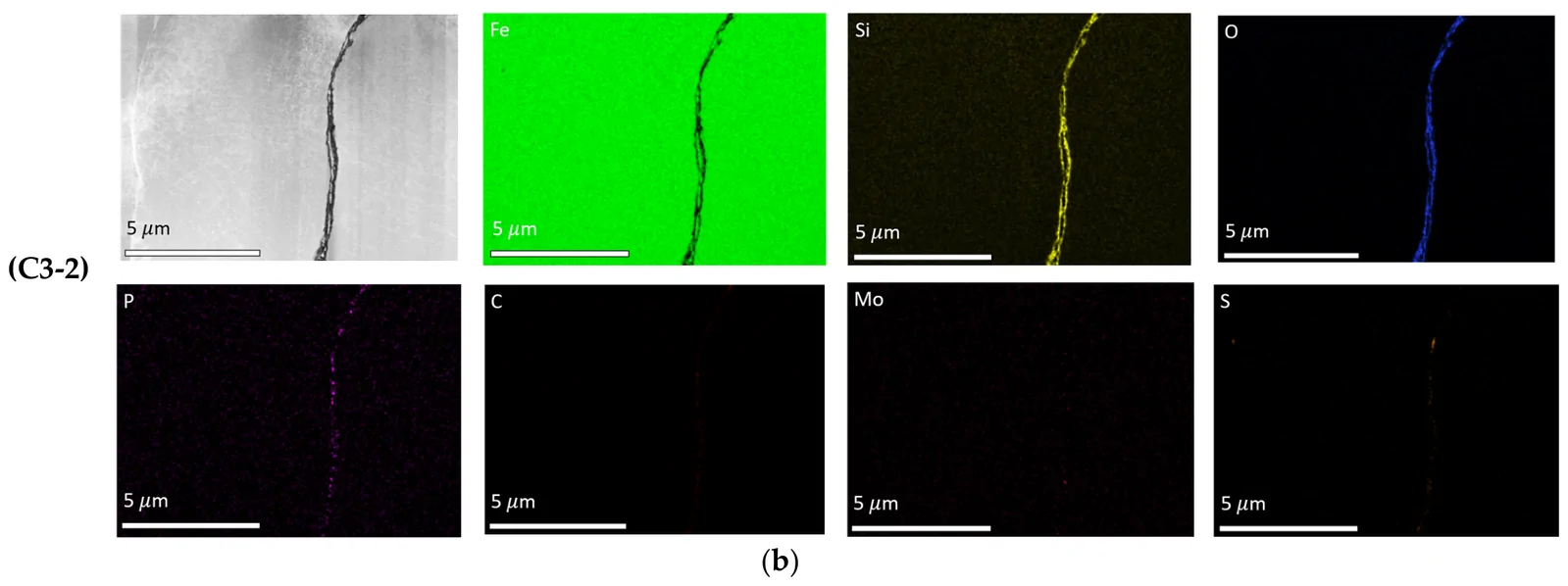
TEM images and EDS elemental mappings of Fe, Si, O, P, C, Mo, and S at the interparticle interfaces of the C3 case: (**a**) C3-1 and (**b**) C3-2. The C3 composition contains 1.0 wt.% MoS_2_ and 0.3 wt.% graphite. The suffixes -1 and -2 indicate TEM lamellae prepared from two different specimens of the same case. Scale bars: 5 μm.

Figure 8 shows the TEM–EDS mapping results obtained at the interparticle interfaces of C3-1 and C3-2. The distributions of Fe and Si were used to distinguish the Fe–Si matrix particles from the interparticle interfaces, and the spatial distributions of P, O, Mo, S, and C were used to compare the distributions of additive-related elements within the interfacial regions [7,15,36,38,39,40].

In C3-1 and C3-2, P and O were distributed together along the relatively thin interfacial regions between the Fe–Si particles. Because P was introduced during the H_3_PO_4_ treatment process, the interfacial distributions of P and O represent the spatial distribution of P–O-containing interfacial components associated with H_3_PO_4_ treatment [6,7,15,27,40,41]. At some interfaces, regions in which the Si and O signals spatially overlapped were also identified, and local interfacial regions in which Si and O were distributed together were observed [7,27,36,40,41].

Mo and S were also distributed at similar locations along the interparticle interfaces of C3. Mo and S are elements derived from the added MoS_2_, and regions in which both elements appeared together indicate the distribution of Mo- and S-containing interfacial components associated with MoS_2_ addition [24,29,31]. The C signal was also identified along the interparticle interfaces. Because the specimens used in this study contained both graphite and polyimide, which contain C, the C signal was analyzed as indicating the distribution of C-containing interfacial regions associated with these carbon-based additives [6,27,29]. The EDS mapping results were compared mainly in terms of the locations and spatial distributions of each element within the interfaces.

The thermal stability of MoS_2_ during heat treatment should also be considered. Previous research on Fe–MoS_2_ systems has shown that Fe sulfides can be thermodynamically more stable than MoS_2_ under iron-based sintering conditions and that graphite addition can retard the reaction between MoS_2_ and Fe [31]. In the present study, TEM–EDS mapping after heat treatment at 720 °C for 30 min showed the local co-distribution of Mo and S at the interparticle interfaces, which was interpreted as the distribution of Mo- and S-containing interfacial components associated with the initial MoS_2_ addition.

In the TEM–EDS results of A3 and B3 presented in Supplementary Materials S2, P and O were also distributed along the interparticle interfaces, and Mo and S appeared at similar locations at several interfaces. In B3-2, a relatively wide C-containing region was observed together with a locally expanded interfacial region. Because the graphite content of B3 was the same as that of A3 and C3 at 0.3 wt.%, this wide C distribution was observed as a local concentration of components associated with carbon-based additives at the corresponding interparticle interface rather than as a result of an increase in graphite content. In addition, Mo and S were distributed together at several locations within the wide interfacial region of B3-2, showing that the local distribution of additive-related components was not uniform depending on the interface location [15,24,29,31].

In C5 and C8, P and O were also distributed along the interparticle interfaces, and Mo and S were present at similar locations in several interfacial regions. In particular, in C5-2, the Mo and S signals were concentrated together in a specific local region. This indicates that Mo- and S-containing components associated with MoS_2_ addition were locally concentrated at some interfaces [24,29,31]. In C8-2, a relatively wide C-containing region was observed together with a locally expanded interfacial region, showing a distribution in which C components associated with carbon-based additives were spread across a wide interfacial region [27,29].

As described above, the interfacial region thickness and elemental distributions observed by TEM–EDS did not show simple linear or monotonic changes with increasing MoS_2_ or graphite content. Rather, even at the same lubricant content, differences were observed in the local distribution of additive-related components and in the morphology and thickness of the interfacial regions depending on the interface location. This indicates that the lubricant content defined at the bulk level does not correspond one-to-one with the actual local interparticle interface condition [7,15,36,39,40,41].

The TEM–EDS results of C3 were interpreted not as independent evidence that alone determines the overall interface condition or electromagnetic properties of C3, but as local microstructural characteristics considered together with the FEA, SEM, and electromagnetic property results [36,39,40,41]. These local interface characteristics were also found to be consistent with the other analysis results.

## 5. Conclusions

This study investigated the effects of combined MoS_2_ and graphite lubricant contents on the compaction characteristics, internal relative-density uniformity, electromagnetic properties, and interparticle microstructure of Fe–5.0 wt.%Si SMC toroidal cores [5,15]. Nine lubricant compositions were evaluated under identical 2P1A processing conditions [5]. FEA was used to evaluate the final compaction state and internal relative-density distribution, while the electromagnetic performance of the fabricated cores was characterized in terms of P_cv_, P_cm_, and H_c_. In addition, SEM and TEM–EDS analyses were performed to examine the interparticle contact state, interfacial region morphology, and local elemental distributions. These results were considered together to evaluate the influence of lubricant composition from compaction, electromagnetic, and microstructural perspectives.

Among the nine lubricant compositions, C3, containing 1.0 wt.% MoS_2_ and 0.3 wt.% graphite, showed the most favorable combined performance. Under the common load-stop conditions, C3 exhibited the smallest relative-density Max–Min of 0.439 and the highest relative-density Min of 0.558 in the FEA, indicating the most uniform internal relative-density distribution among the investigated cases. At a maximum magnetic flux density of 300 mT, C3 also exhibited the lowest P_cv_, P_cm_, and H_c_ at both 1000 and 1500 Hz. The P_cv_ values were 113.50 and 173.70 kW/m^3^, the P_cm_ values were 15.54 and 23.78 W/kg, and the H_c_ values were 134.83 and 137.40 A/m at 1000 and 1500 Hz, respectively. SEM observations further showed relatively continuous interparticle contact throughout the Top, Middle, and Bottom positions of C3, while TEM–EDS showed thin and similar interfacial regions in two different C3 specimens, with mean thicknesses of 99.9 and 89.9 nm. Thus, the favorable internal relative-density uniformity, low magnetic loss, and comparatively consistent interparticle microstructure were observed together for the C3 composition.

From an engineering perspective, these results provide quantitative reference values for lubricant selection in Fe–5.0 wt.%Si SMC toroidal cores manufactured under the present 2P1A conditions. In particular, the C3 composition combined a relative-density Max–Min of 0.439 with P_cv_ values of 113.50 and 173.70 kW/m^3^ at 1000 and 1500 Hz, respectively. Therefore, within the investigated material, lubricant-composition, and processing ranges, the combination of 1.0 wt.% MoS_2_ and 0.3 wt.% graphite can be considered an appropriate lubricant condition for Fe–5.0 wt.%Si SMCs when internal relative-density uniformity and electromagnetic loss characteristics are considered together.

However, C3 should not be regarded as a universally optimal lubricant composition for all SMC systems. The present conclusions were derived specifically for Fe–5.0 wt.%Si powder, the investigated MoS_2_/graphite composition range, the toroidal geometry, and the applied 2P1A processing conditions. Variations in alloy composition, powder characteristics, compact geometry, or processing conditions may alter the effects of the lubricant composition. Accordingly, the C3 condition should be interpreted as the most suitable composition within the scope of the present Fe–5.0 wt.%Si SMC study rather than as a general design criterion applicable to all SMC materials.

Future work will investigate the effects of powder particle size on compaction behavior, internal relative-density uniformity, microstructure, and electromagnetic properties to determine how particle-size variation influences the relationships identified in the present study [25]. In addition, dynamic fatigue strength testing under cyclic mechanical loading should be performed to evaluate the long-term mechanical durability and structural reliability of the composite and to extend the present evaluation toward service-relevant operating conditions.

## Figures and Tables

**Table 1:** Lubricant and binder composition conditions of the Fe–5.0 wt.%Si powder mixtures.

Case	MoS_2_ (wt.%)	Graphite (wt.%)	H_3_PO_4_ (wt.%)	Polyimide (wt.%)
A3	0.5	0.3	0.25	0.25
A5	0.5	0.5	0.25	0.25
A8	0.5	0.8	0.25	0.25
B3	0.75	0.3	0.25	0.25
B5	0.75	0.5	0.25	0.25
B8	0.75	0.8	0.25	0.25
C3	1.0	0.3	0.25	0.25
C5	1.0	0.5	0.25	0.25
C8	1.0	0.8	0.25	0.25

**Table 2:** Material parameters and constitutive settings used in the FEA of the Fe–5.0 wt.%Si powder compact.

Parameter	Value
Initial relative density	0.428
Mechanical formulation	Powder-type rigid-plastic
Porous yield model	Default Oyane porous material model
Hardening law	Isotropic hardening
Flow-stress definition	$\bar{\sigma }=\bar{\sigma }(\bar{\varepsilon },\dot{\bar{\varepsilon }},T)$
Effective strain range	0–0.45 (6 input points)
Effective strain rate	0.1 s^−1^
Temperature range	20–902 °C (10 input points)

**Table 3:** Powder–die friction coefficients applied to the nine lubricant compositions during the first- and second-pressing stages [5].

Case	RT	150 °C	350 °C	Extrapolated 550 °C
A3	0.507	0.777	0.664	0.825221
A5	0.758	0.815	0.71	0.692652
A8	0.818	0.796	0.548	0.436991
B3	0.819	0.811	0.609	0.51597
B5	0.841	0.743	0.568	0.448048
B8	0.985	0.753	0.753	0.62174
C3	0.846	0.751	0.509	0.376875
C5	0.829	0.708	0.743	0.681924
C8	0.795	0.85	0.827	0.854322

**Table 4:** Comparison of the experimental average heights and FEA-predicted final heights of the Fe–5.0 wt.%Si toroidal specimens.

Case	Average Height (mm)	FEA-Predicted Final Height (mm)	Absolute Difference (mm)	Relative Error (%)
A3	4.02	4.03	0.01	0.25
A5	4.01	4.01	0.00	0.00
A8	4.00	4.00	0.00	0.00
B3	4.01	3.98	0.03	0.75
B5	4.00	4.01	0.01	0.25
B8	4.04	3.97	0.07	1.73
C3	3.99	4.00	0.01	0.25
C5	4.01	4.01	0.00	0.00
C8	4.03	3.99	0.04	0.99

**Table 5:** Two-dimensional pore area fractions obtained from digital image analysis of SEM micrographs at the Top, Middle, and Bottom positions of the Fe–5.0 wt.%Si toroidal compacts.

Case	Top (%)	Middle (%)	Bottom (%)
A3	2.634	2.303	3.161
A5	2.285	3.576	2.694
A8	7.205	2.661	2.628
B3	5.620	3.915	2.808
B5	2.081	4.616	2.674
B8	3.151	2.717	1.659
C3	1.856	1.945	2.827
C5	4.670	3.628	3.727
C8	4.385	5.272	4.220

**Table 6:** Interfacial region thickness measured from TEM images for the A3, B3, C3, C5, and C8 cases.

Case	Lamella	Minimum Thickness (nm)	Maximum Thickness (nm)	Mean Interfacial Region Thickness (nm)
A3	A3-1	122	561	223.7
	A3-2	373	1130	678.5
B3	B3-1	89	1580	474.9
	B3-2	461	2400	1426.1
C3	C3-1	43	181	99.9
	C3-2	51	152	89.9
C5	C5-1	173	526	351.7
	C5-2	129	220	163.5
C8	C8-1	195	507	339.8
	C8-2	216	1290	671.8

## Data Availability

The original contributions presented in the study are included in the article/Supplementary Materials; further inquiries can be directed to the corresponding author.

## Supplementary Materials

The following supporting information can be downloaded at: https://www.mdpi.com/article/10.3390/ma19204311/s1, Supplementary_S1_Fe5.0wtSi_Electromagnetic_Data: Frequency-dependent inductance, permeability, and Q-value data of the nine Fe–5.0 wt.%Si toroidal cores; Supplementary_S2_Fe5.0wtSi_TEM-EDS_Mapping: TEM images and EDS elemental mappings of Fe, Si, O, P, C, Mo, and S at the interparticle interfaces of the A3, B3, C5, and C8 cases. Figure S1: TEM images and EDS elemental mappings of Fe, Si, O, P, C, Mo, and S at the interparticle interfaces of the A3 case: (a) A3-1 and (b) A3-2. The A3 composition contains 0.5 wt.% MoS_2_ and 0.3 wt.% graphite. The suffixes -1 and -2 indicate TEM lamellae prepared from two different specimens of the same case. Scale bars: 5 μm.; Figure S2: TEM images and EDS elemental mappings of Fe, Si, O, P, C, Mo, and S at the interparticle interfaces of the B3 case: (a) B3-1 and (b) B3-2. The B3 composition contains 0.75 wt.% MoS_2_ and 0.3 wt.% graphite. The suffixes -1 and -2 indicate TEM lamellae prepared from two different specimens of the same case. Scale bars: 5 μm.; Figure S3: TEM images and EDS elemental mappings of Fe, Si, O, P, C, Mo, and S at the interparticle interfaces of the C5 case: (a) C5-1 and (b) C5-2. The C5 composition contains 1.0 wt.% MoS_2_ and 0.5 wt.% graphite. The suffixes -1 and -2 indicate TEM lamellae prepared from two different specimens of the same case. Scale bars: 5 μm.; Figure S4: TEM images and EDS elemental mappings of Fe, Si, O, P, C, Mo, and S at the interparticle interfaces of the C8 case: (a) C8-1 and (b) C8-2. The C8 composition contains 1.0 wt.% MoS_2_ and 0.8 wt.% graphite. The suffixes -1 and -2 indicate TEM lamellae prepared from two different specimens of the same case. Scale bars: 5 μm.
