# Supplementary material for: Evaluation of Electromagnetic Properties of Fe–5.0 wt.%Si According to MoS2 and Graphite Lubricant Contents
Source: Materials (Basel). 2026 Oct 13;19(20):4311. doi: 10.3390/ma19204311 (PMC13707035; doi:10.3390/ma19204311)
Supplement: Supplementary file 1 [file materials-19-04311-s001.zip › Supplementary_Materials_Fe5.0wtSi/Supplementary_S2_Fe5.0wtSi_TEM-EDS_Mapping.pdf]

(A3-1)

(A3-2)

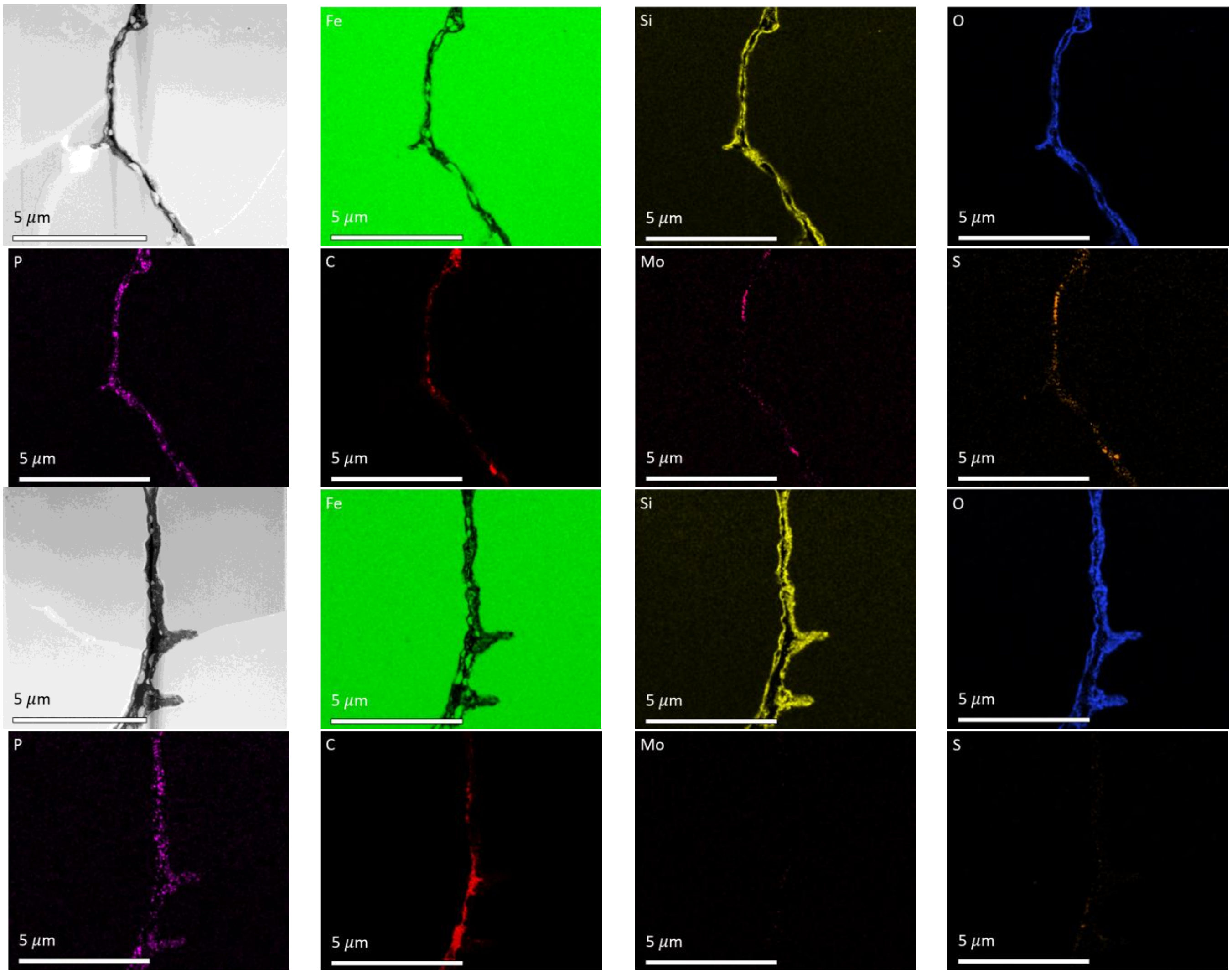

Figure S1. TEM images and EDS elemental mappings of Fe, Si, O, P, C, Mo, and S at the interparticle interfaces of the A3 case: (a) A3-1 and (b) A3-2. The A3 composition contains 0.5 wt.% MoS_2 and 0.3 wt.% graphite. The suffixes -1 and -2 indicate TEM lamellae prepared from two different specimens of the same case. Scale bars: 5 µm.

(B3-1)

(B3-2)

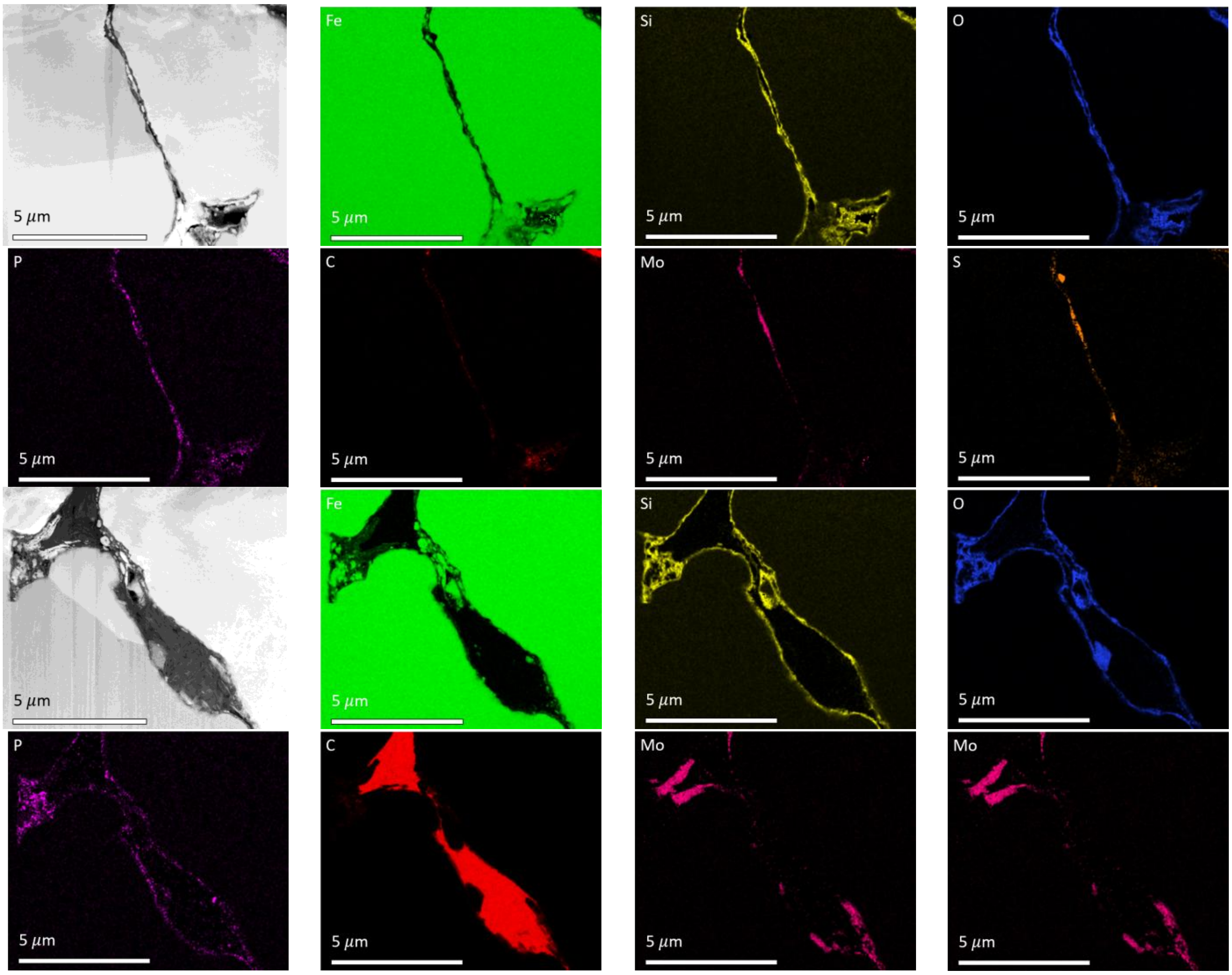

Figure S2. TEM images and EDS elemental mappings of Fe, Si, O, P, C, Mo, and S at the interparticle interfaces of the B3 case: (a) B3-1 and (b) B3-2. The B3 composition contains 0.75 wt.% MoS_2 and 0.3 wt.% graphite. The suffixes -1 and -2 indicate TEM lamellae prepared from two different specimens of the same case. Scale bars: 5 μm.

(C5-1)

(C5-2)

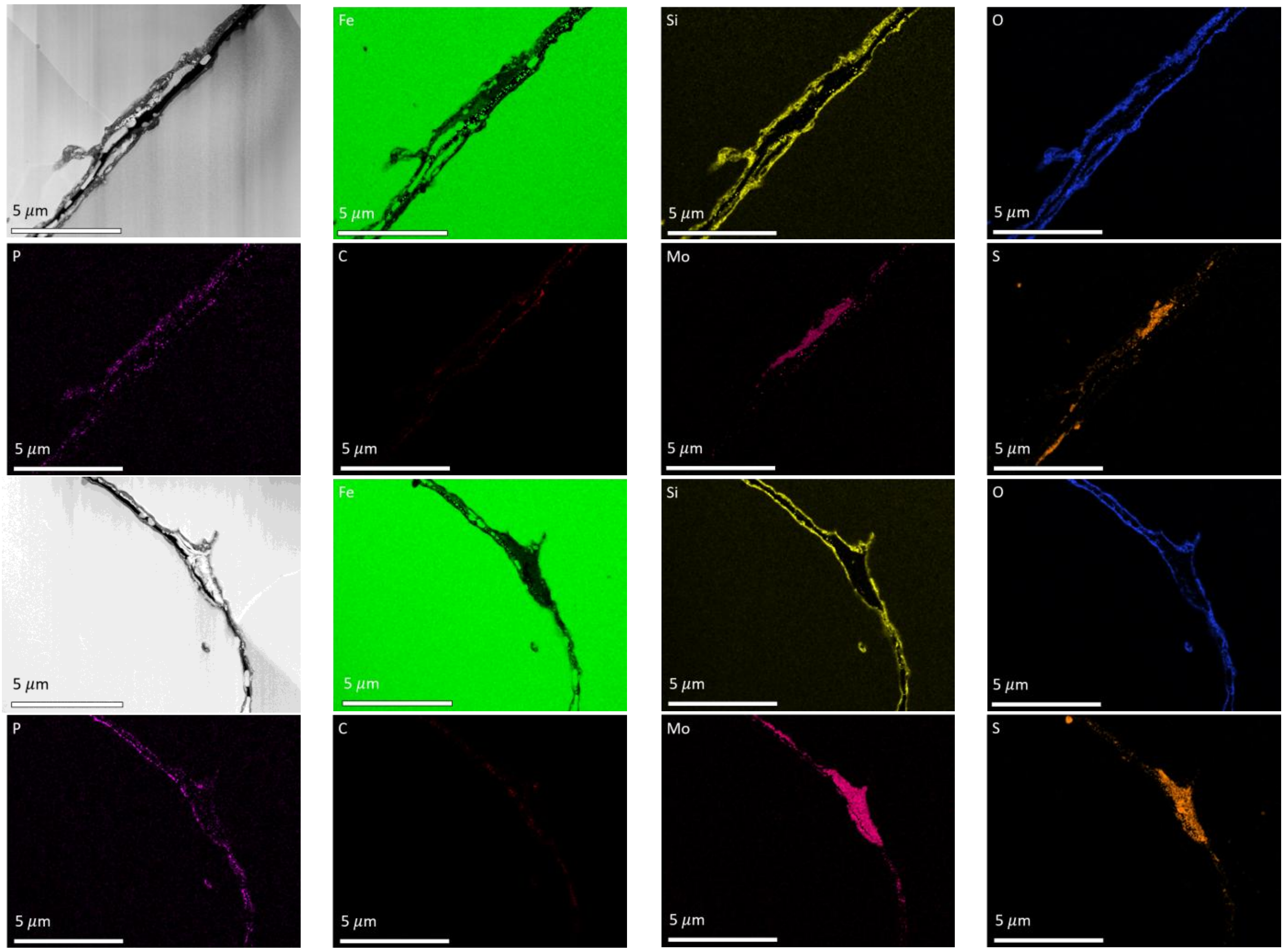

Figure S3. TEM images and EDS elemental mappings of Fe, Si, O, P, C, Mo, and S at the interparticle interfaces of the C5 case: (a) C5-1 and (b) C5-2. The C5 composition contains 1.0 wt.% MoS_2 and 0.5 wt.% graphite. The suffixes -1 and -2 indicate TEM lamellae prepared from two different specimens of the same case. Scale bars: 5 μm.

(C8-1)

(C8-2)

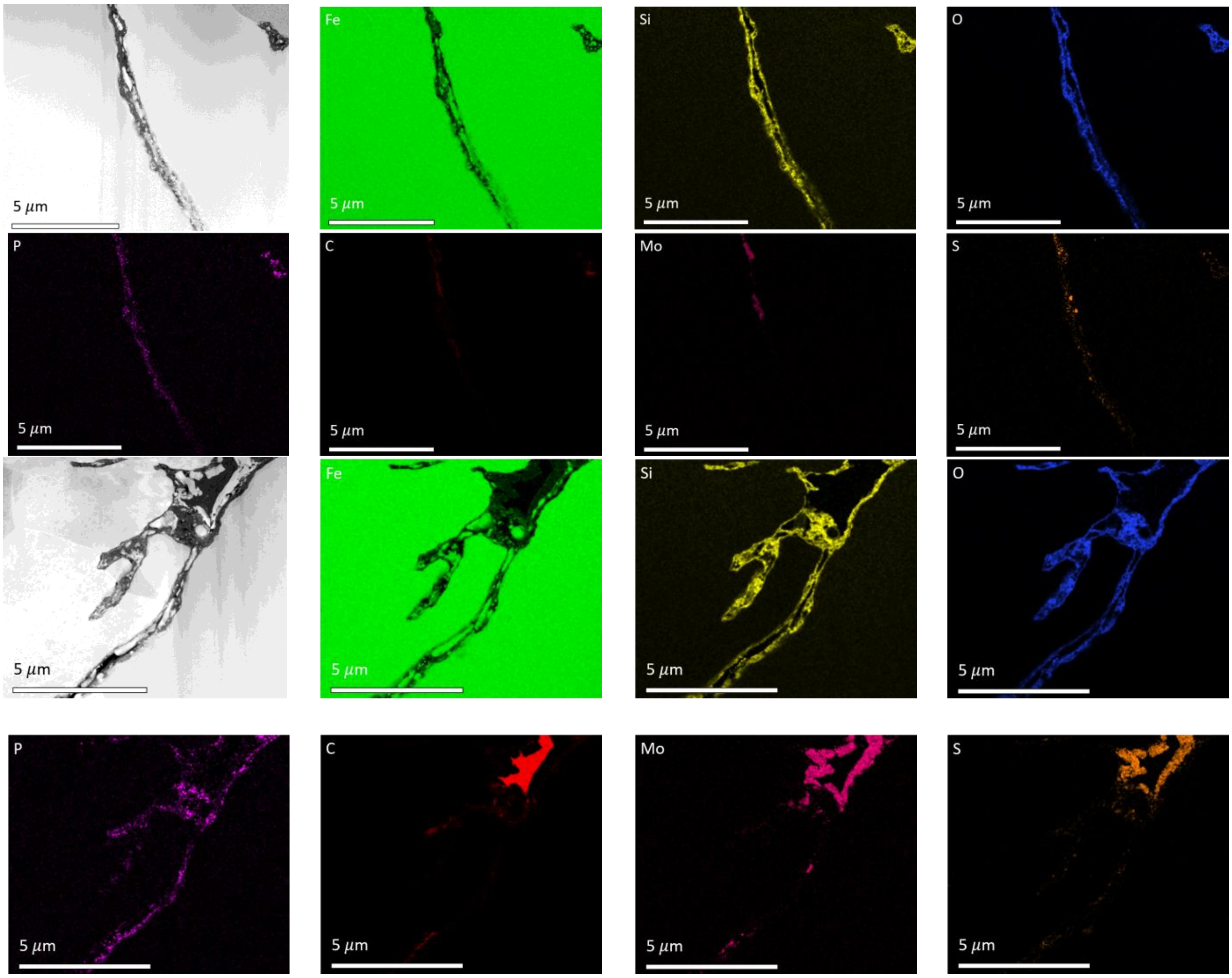

Figure S4. TEM images and EDS elemental mappings of Fe, Si, O, P, C, Mo, and S at the interparticle interfaces of the C8 case: (a) C8-1 and (b) C8-2. The C8 composition contains 1.0 wt.% MoS_2 and 0.8 wt.% graphite. The suffixes -1 and -2 indicate TEM lamellae prepared from two different specimens of the same case. Scale bars: 5 μm.
